# COVID-19 and the Endocrine System: A Comprehensive Review on the Theme

**DOI:** 10.3390/jcm10132920

**Published:** 2021-06-29

**Authors:** Giuseppe Lisco, Anna De Tullio, Assunta Stragapede, Antonio Giovanni Solimando, Federica Albanese, Martina Capobianco, Vito Angelo Giagulli, Edoardo Guastamacchia, Giovanni De Pergola, Angelo Vacca, Vito Racanelli, Vincenzo Triggiani

**Affiliations:** 1 Interdisciplinary Department of Medicine, Section of Internal Medicine, Geriatrics, Endocrinology and Rare Diseases, School of Medicine, University of Bari “Aldo Moro”, 70124 Bari, Italy; giuseppe.lisco@uniba.it (G.L.); annadetullio16@gmail.com (A.D.T.); vitogiagulli58@gmail.com (V.A.G.); edoardo.guastamacchia@uniba.it (E.G.); vincenzo.triggiani@uniba.it (V.T.); 2 Department of Biomedical Sciences and Human Oncology, Section of Internal Medicine “G. Baccelli”, University of Bari School of Medicine, 70124 Bari, Italy; assunta-s@live.it (A.S.); antonio.solimando@uniba.it (A.G.S.); federica.albanese94@gmail.com (F.A.); martina.capobianco22@gmail.com (M.C.); angelo.vacca@uniba.it (A.V.); 3 Department of Biomedical Sciences and Human Oncology, Section of Internal Medicine and Clinical Oncology, University of Bari Aldo Moro, 70124 Bari, Italy; giovanni.depergola@uniba.it; 4 National Institute of Gastroenterology “Saverio de Bellis”, Research Hospital, 70013 Castellana Grotte, Italy

**Keywords:** SARS-CoV-2, COVID-19, pituitary, thyroid, parathyroid, vitamin D, hypercalcemia, osteoporosis, adrenal gland, Addison disease, Cushing syndrome, review

## Abstract

Background and aim. The review aimed to summarize advances in the topic of endocrine diseases and coronavirus disease 2019 (COVID-19). Methods. Scientific and institutional websites and databases were searched and data were collected and organized, when plausible, to angle the discussion toward the following clinical issues. (1) Are patients with COVID-19 at higher risk of developing acute or late-onset endocrine diseases or dysfunction? (2) May the underlying endocrine diseases or dysfunctions be considered risk factors for poor prognosis once the infection has occurred? (3) Are there defined strategies to manage endocrine diseases despite pandemic-related constraints? Herein, the authors considered only relevant and more frequently observed endocrine diseases and disorders related to the hypothalamic-pituitary region, thyroid and parathyroid glands, calcium-phosphorus homeostasis and osteoporosis, adrenal glands, and gonads. Main. Data highlight the basis of some pathophysiological mechanisms and anatomical alterations of Severe Acute Respiratory Syndrome Coronavirus 2 (SARS-CoV-2)-induced endocrine dysfunctions. Some conditions, such as adrenal insufficiency and cortisol excess, may be risk factors of worse clinical progression once the infection has occurred. These at-risk populations may require adequate education to avoid the SARS-CoV-2 infection and adequately manage medical therapy during the pandemic, even in emergencies. Endocrine disease management underwent a palpable restraint, especially procedures requiring obligate access to healthcare facilities for diagnostic and therapeutic purposes. Strategies of clinical triage to prioritize medical consultations, laboratory, instrumental evaluations, and digital telehealth solutions should be implemented to better deal with this probably long-term situation.

## 1. SARS-CoV-2 and Coronavirus Disease 2019

Coronavirus disease 2019 (COVID-19) is a highly transmissible infectious disease caused by the Severe Acute Respiratory Syndrome Coronavirus (SARS-CoV) 2 (SARS-CoV-2), a positive-sense, single-strand, enveloped RNA virus belonging to the family of coronaviridae [1,2]. First metagenomic RNA sequencing of SARS-CoV-2 showed the single-strand RNA was closely related to bat SARS-like coronaviruses (89%) [3], while it was distant from SARS-CoV (79%) and the Middle East Respiratory Syndrome Coronavirus (MERS-CoV) (50%) [4]. Phylogenetic analysis suggested that SARS-CoV-2 progenitors circulated in animal hosts and underwent a naturally occurring selection before the zoonotic spillover and, lastly, adapting to infect humans [5,6,7]. SARS-CoV-2 transmission occurs through the airways. Droplets may be the main transmission route, even if a relevant concentration of viral progenies was found in tiny particles as breathable aerosol [8]. The transmission risk increases after unaware and prolonged exposure to “spreaders”, being more considerable indoors or in poorly ventilated places than outdoors [9]. After exposure, the individual enters the asymptomatic phase, during which SARS-CoV-2 replicates considerably. Asymptomatic infection occurs in around 16% of the cases in adults and 28% in children [10], the main reservoir of asymptomatic SARS-CoV-2 infection [11]. Around one out of two initially asymptomatic patients can develop symptoms shortly after [10]. These patients have a pre-symptomatic phase during which viral spread from airways becomes consistent 1 to 3 days before symptoms’ onset [12] and might transmit SARS-CoV-2 with high probability [13].

The acute infection occurs asymptomatically or with mild to moderate symptoms (80%), such as fever, pharyngodynia, asthenia, myalgias, hypo- or anosmia, and dysgeusia [14]. Severe and critical clinical progression was reported in 15% and 5% of the cases, respectively [14]. Elderly patients [15] and those with underlying comorbidities, including arterial hypertension [16], obesity [17,18], poorly controlled diabetes mellitus [19], active malignancy [20], chronic obstructive pulmonary disease [21], and chronic renal and cardiovascular diseases [22], are prone to develop a worse clinical progression [23]. Severe and critical cases usually exhibit a high in-hospital mortality rate and the death occurs because of acute pulmonary distress, acute cardiovascular and renal injury, sepsis, and multi-organ failure [24,25,26,27]. Post-acute COVID-19 is a composite clinical syndrome that has been described among patients who recovered from the acute infection incompletely. Symptoms of post-acute COVID-19 may include persistent asthenia and usually cough, fatigue, dyspnea or breathlessness, and memory and concentration deficits. This clinical condition is usually accompanied by laboratory and instrumental pulmonary, cardiovascular, renal, coagulative, and neurological sequelae [28].

## 2. The Endocrine System and COVID-19

SARS-CoV-2 enters host cells mainly through angiotensin-converting enzyme 2 (ACE2) and transmembrane protease serine 2 (TMPRSS2), well-recognized viral receptors [29]. In the respiratory tract, SARS-CoV-2 infects ciliated, mucus-secreting, and Clara cells in the bronchial epithelium and type 1 pneumocyte in the lung [30]. However, as SARS-CoV-2 exhibits a wide organotropism, it may affect other tissues, precipitating pre-existing conditions [31].

Recent advances suggest a possible infection of the endocrine system in COVID-19 patients [32]. However, the pathophysiological characterization and clinical relevance of this damage and the impact of related endocrine dysfunction on prognosis are still not completely understood. ACE2 and TMPRSS2 are expressed in several endocrine tissues, namely, the hypothalamus, pituitary, thyroid, adrenal, gonads, and pancreatic islets [33]. As the endocrine system is an issue during the COVID-19 pandemic, several clinical questions are necessary. Specifically, it should be clarified whether (1) patients with COVID-19 could be at higher risk of developing acute or late-onset endocrine diseases or dysfunction; (2) underlying endocrine diseases or dysfunctions could be risk factors for poor prognosis once the infection occurred; (3) pandemic-related community and healthcare service restrictions and reorganization may contribute to change the epidemiology of endocrine diseases or dysfunctions or affect their management. Accordingly, the authors searched PubMed/MEDLINE, Web of Science, Scopus, Cochrane Library, Google scholar, and institutional websites for free-text words and medical subject headings’ terms related to “coronavirus”, “sars cov 2”, “covid 19”, “hypopituitarism”, “cushing’s disease”, “cushing’s syndrome”, “acromegaly”, “growth hormone deficiency”, “diabetes insipidus”, “inappropriate antidiuretic hormone secretion syndrome”, “hyponatremia”, “hypernatremia”, “hypothyroidism”, “hyperthyroidism”, “thyroid nodule”, “thyroid carcinoma”, “hyperparathyroidism”, “hypoparathyroidism”, “hypercalcemia”, “hypocalcemia,” “vitamin D”, “hypercortisolism”, “addison’s disease”, “estrogen”, “testosterone”, “polycystic ovary syndrome”, “male hypogonadism”, “immune system”, and “thrombosis”. Original studies, reviews, systematic reviews, and meta-analyses written in English were searched and selected, focusing on the following topics: the pathophysiology of endocrine dysfunction possibly related to SARS-CoV-2 infection; relationship between pre-existing endocrine dysfunction and worse clinical progression of COVID-19 from hospitalized patients and deaths; brief analysis of challenges affecting endocrine care and strategies to improve endocrine diseases’ management during the pandemic.

## 3. Hypothalamus and Pituitary Gland

Hypothalamic and pituitary tissues express ACE2 and may be a target of SARS-CoV-2 [34]. The main associated hormonal dysfunctions after 3 months from recovery of previous coronaviruses (e.g., SARS-CoV) infections were central hypocortisolism (39%) and central hypothyroidism (5%) [35]. Hypothalamic and pituitary involvement in patients with SARS has been demonstrated from autoptic studies. As an example, Wei et al. examined pituitary autoptic specimens from four men and one woman with SARS. They found hypophyseal somatotroph, thyrotrope, and corticotrope-producing cells were reduced in number and showed changes suggestive of acute injuries, such as edema and neuronal degeneration. These findings were consistent with the serological evidence of reduced circulating levels of growth hormone (GH), thyroid-stimulating hormone (TSH), and adrenocorticotropic hormone (ACTH) [36].

SARS-CoV and SARS-CoV-2 express specific amino acid sequences exhibiting a remarkable homology to ACTH residues. It has been hypothesized that host response against SARS-CoV (and SARS-CoV-2) may lead to the production of cross-reacting antibodies inactivating or destroying the endogenous ACTH [34,37]. It could be an adaptive mechanism by which the virus escapes to ACTH response, as an immune-invasive strategy aiming to knock down the host’s cortisol response [38]. On the other hand, this mechanism could be a trigger factor for an occurring secondary adrenal insufficiency. However, this hypothesis is hindered from being confirmed or excluded, as ACTH and cortisol levels are usually not assessed routinely in patients with SARS or COVID-19. Accordingly, the use of corticosteroids in all the COVID-19 patients is not recommended [39]. On the one hand, they significantly attenuate or prevent a consistent pulmonary injury in patients with COVID-19 pneumonia [40] and reduce all-cause mortality in critically ill patients [41]. This could be attributable to a combined effect of glucocorticoids in orchestrating anti-inflammation and enhancing tissue repair by modulating the crucial activity of macrophages and monocytes [42]. On the other hand, corticosteroids may hamper the immune response against viral infections in the respiratory tract, especially at the initial stage [43]. This fact could result in a prolonged viral shedding as the consequence of a blunted host’s immune response against SARS-CoV-2 (e.g., methylprednisolone) [44]. Additionally, glucocorticoids induce adverse effects especially when administered at high-dose regimens [45]. The most common adverse events include signs or related hypercortisolism symptoms (e.g., hypertension, hyperglycemia), possibly complicating clinical management of COVID-19 [46,47]. Therefore, high-dose corticosteroid (e.g., dexamethasone 6 mg/day intravenously or orally per 10 consecutive days) treatment is indicated in severe COVID-19 cases exhibiting exaggerated immune response and pulmonary injury [40]. As another issue, abrupt discontinuation of glucocorticoid treatment may induce a secondary adrenal insufficiency, even manifesting with adrenal crisis. The probability of observing adrenal insufficiency after glucocorticoid discontinuation in chronically treated patients increases with specific routes of administration (intra-articular) and considering the cumulative dose (>20 mg prednisone daily or equivalents) and time of exposure (more than 3 weeks) [48]. However, it should be noted that the risk of adrenal insufficiency after glucocorticoid withdrawal is unpredictable. Therefore, it is essential to provide a cautious glucocorticoid tapering before discontinuation [49].

Patients with adrenal insufficiency who are still on glucocorticoid replacement should be advised to supplement hydrocortisone therapy once a diagnosis of COVID-19 is confirmed. When signs and symptoms of infection occur (e.g., fever), patients should refer to the so-called “sick day rule”. Adequate education or reinforcing are essential to avoid concerns [50].

Post-COVID-19 cases of central diabetes insipidus have been described. It is believed to be the consequence of a direct or immune-mediated (neuro)hypophysitis [51] or due to hypoxic encephalopathy, especially among patients who experienced serious respiratory failure [52]. On the other hand, nephrogenic diabetes insipidus may be the consequence of sedation (i.e., sevoflurane) in critically ill patients who underwent endotracheal intubation [53]. In this clinical scenario, an adequate hypotonic fluid replacement should be administered intravenously, followed by desmopressin supplementation [54]. The plasmatic sodium concentration and 24-h diuresis should be monitored for evaluating treatment effectiveness [54]. 

Patients with a pre-existing diabetes insipidus and with established medical treatment may develop dilutional hyponatremia as a common adverse effect of chronic desmopressin supplementation (mild hyponatremia in 27%, severe in 15%). To prevent hyponatremia, a regular check of serum electrolytes and periodic medical consultations are essential. Restricted accessibility to blood testing and medical appointments during the pandemic may hamper the management of these patients. When these drawbacks are factual, patients should be educated to perform a periodic and intermittent withdrawal of desmopressin administration (e.g., 1 day a week) to achieve regular phases of aquaresis and polyuria [55]. Hypernatremia could be observed in COVID-19 patients admitted to intensive care units. This is attributable to excessive insensible water loss due to fever, high respiration rate, or diuretics inadequately replaced by fluid intake (e.g., loss of cognition, endotracheal intubation). Patients with diabetes insipidus may be at higher risk of hypernatremia in case of fever. Moreover, intranasal desmopressin absorption may be reduced in mild COVID-19 patients with cold symptoms, thus becoming another mechanism of hypernatremia. In this case, oral desmopressin should be more appropriate unless possible drawbacks (vomiting), in which quick-shifting to intravenous desmopressin is mandatory. When hypernatremia occurs, clinicians should monitor urinary osmolarity and volume, plasma sodium concentration, and osmolarity as fast as every 2 to 4 h until plasmatic sodium returns to safe levels [55]. Severe hypernatremia may induce a hypercoagulable state, as the production of von Willebrand factor from endothelial cells is enhanced by extracellular sodium retention (animal models) [56]. It should be considered that desmopressin, per se, has pro-coagulative properties since it may increase the synthesis of von Willebrand factor and factor VIII of coagulative cascade [57]. Given this assumption, patients with diabetes insipidus should be adequately managed to prevent possible hydro-electrolytic imbalance, especially in the case of COVID-19. 

Oxytocin is released from the neuro-hypophysis and plays a role in regulating several functions, mainly at central nervous and cardiovascular systems [58]. Additionally, oxytocin reduces inflammation and oxidative stress reactions by decreasing cytokines released from activated macrophages. This was proposed as a possible defense mechanism against COVID-19 [59]. Furthermore, activation of the oxytocin receptor, also expressed in the pulmonary artery, can produce a vasodilatory effect by enhancing the nitroxide pathway [59]. Moreover, oxytocin has been demonstrated to be a natural inhibitor of dipeptidyl-peptidase IV and may have a potential role in contrasting coronaviruses’ internalization into host cells [60].

Patients with new-onset or uncontrolled Cushing disease (CD) may be at higher risk of infections because of immune suppression, and the risk of mortality is increased, primarily due to cardiovascular diseases [61,62]. Therefore, the risk of poor outcomes in the case of COVID-19 may be a clinical concern, mainly when hypercortisolism is weakly controlled. CD is a rare condition, and a few cases of COVID-19 in those patients have been described [63]. Recently, a Russian experience in managing three patients with CD and confirmed SARS-CoV-2 infection has been published [64]. Although data are too scanty to confirm the structurally consistent hypothesis, the authors found a worse prognosis in a 71-year-old woman with newly diagnosed CD who died due to bilateral hemorrhagic pneumonia and a better outcome in a 38-year-old woman with a 5-year medical history of active CD who fully recovered after 24 days of oxygen and antibiotic therapy. Last, a 66-year-old woman with a 4-year medical history of mild CD remained asymptomatic [64]. Moreover, adrenal insufficiency may complicate the clinical progression in COVID-19, as described in a case report [65]. In addition, it could be of help to consider drug interaction among ongoing medications to control cortisol excess (somatostatin analogues, cabergoline, adrenal steroidogenesis blockers, glucocorticoid receptor antagonists) and contingent COVID-19 medications. The leading warnings for possible interactions are essentially related to hypokalemia, QT interval prolongation, and hypoglycemia. Therefore, caution should be used when administering hydroxychloroquine, ritonavir, and azithromycin and patients should be adequately monitored during the treatment. Additionally, the use of systemic glucocorticoids, such as dexamethasone, in patients taking mifepristone, a glucocorticoid receptor antagonist, may be challenging as the latter reduces the efficacy of the former [66]. Tocilizumab, a fully humanized monoclonal antibody blocking the IL-6 receptor, may have a rationale in severe COVID-19 cases with relevant pulmonary involvement and systemic inflammation. Since IL-6 down-regulates cytochrome P450s, as observed during systemic inflammation, tocilizumab is an inductor of the cytochrome P450 3A4 activity that is involved in the clearance of a considerable number of medications, including ketoconazole and mifepristone [67]. Hence, a dose adjustment of ketoconazole and mifepristone could be necessary in case of concomitant administration of tocilizumab. Mitotane is a potent inductor of the cytochrome P450 3A4 and a possible interaction with remdesivir is expected. However, as the hepatic clearance accounts for the 10% of the remdesivir global clearance, the abovementioned interaction could have weak clinical consequence in CD [68].

GH-related disorders may have an impact on COVID-19 prognosis. A reduced GH secretion has been observed among COVID-19 patients with worse prognosis even if the phenomenon requires further pathophysiological investigation [69]. Patients with GH deficiency may be exposed to an increased cardio-metabolic risk [70,71] more than the general population. Adults, rather than youngsters, with GH deficiency may be predisposed to poor prognosis once SARS-CoV-2 infection has occurred, particularly in the case of weak disease control (e.g., poor adherence to GH replacement) [69]. Acromegaly leads to an excess of cardiovascular, respiratory, and metabolic risk when the patients fail to control insulin-like growth factor 1 levels despite the treatment [72]. As the abovementioned risk factors may lead to poor prognosis, it is essential to maintain better control of GH disorders, even despite the pandemic-related constraints. In fact, the management of these patients is suffering some drawbacks, as reported by the international ACROCOVID survey [73]. According to the European recommendations, the management of acromegaly and other pituitary diseases should be the same during the pandemic as in other periods but with some adjustments. As an example, unnecessary in-person appointments should be restricted by organizing, when possible, virtual visits and other forms of at-distance consultations. Similarly, the frequency of laboratory, radiological, or visual field assessment should be considered only in selected cases (new-onset, recurrence, or uncontrolled cases) [55]. Medical management could be the preferable approach, as it may safely defer neurosurgery and radiotherapy, especially when neurological compressive effects or impairment in the visual field are not a complaint [74]. However, visual deterioration, headache, and mass enlargement remain the underlying indications for surgical treatment [75]. This is to better deal with the fact that pituitary surgery is usually scheduled as a non-urgent procedure, and the number of pituitary interventions was restricted last year. Moreover, the risk of SARS-CoV-2 transmission during endonasal endoscopic approach from potentially positive patients and the neurosurgery team is elevated [76]. However, more recent experience suggested that an appropriate selection of non-deferrable endonasal endoscopic surgery cases can increase neurosurgery efficiency and safety for patients and healthcare providers [77]. This is feasible after excluding possible SARS-CoV-2-infected cases during pre-operative screening and, as a perspective, after a satisfactory achievement of vaccination coverage of healthcare personnel and patients.

Hypothalamic and pituitary diseases, per se, are not expected to be a risk factor for poor prognosis in SARS-CoV-2 infection. However, when hypopituitarism is the matter, patients may be at risk of poor outcomes, although data are still lacking and risks are difficult to quantify. Reasonably, patients should be educated to carefully manage hormonal replacement therapy regardless of medical consultations, especially in adrenal insufficiency [74]. Glucocorticoid replacement should be increased during fever and symptoms suggestive for COVID-19 (e.g., hydrocortisone 20 mg, 1 tablet every 6 h) to provide a 3-fold increase in the habitual daily dose. To better deal with this issue, hydrocortisone prescription should include extra tablets for emergency use [78]. When clinical deterioration occurs, manifesting with neurological or gastrointestinal symptoms (vomiting, diarrhea), hydrocortisone should be started intravenously even at home (100 mg, vial) and sustained during the hospital stay up to 200 mg/day, continuously or intermittently (e.g., 50 mg every 6 h). Hydro-electrolytic balance and arterial pressure should be sustained by a continuative intravenous saline 0.9% infusion. Gradual steroidal tapering is necessary when clinical conditions improves and oral administration could be restored. As the frequency of follow-up visits and medical consultations could be restricted, therapy adjustments of hormonal replacement therapy could be performed mainly through clinical judgment rather than laboratory investigations [78]. The frequency of reassessments could be set every 6–12 months in most cases, as usual, but intercurrent visits in stable patients could be replaced through telemedicine [74].

## 4. Thyroid Gland

SARS-CoV-2 may lead to thyroid injury and dysfunction [79], as discussed elsewhere [80,81]. As some examples, several subclinical and atypical thyroiditis cases have been described in infected patients [82,83,84,85,86,87,88,89]. The onset is usually described a few weeks after the diagnosis of SARS-CoV-2 infection. The clinical course is usually short and uncomplicated. Euthyroidism could be restored after a short-term oral prednisone therapy [90]. Some cases of autoimmune thyroiditis have also been reported [91,92,93]. In a retrospective observational study among hospitalized patients with COVID-19, hypothyroidism was found in 5% of patients and thyrotoxicosis in 20% of COVID-19 cases. The prevalence of autoimmune thyroiditis was low, and the incidence of possible new-onset cases has not been assessed [94]. Previous experience with other coronaviruses suggested that hypothyroidism, primarily due to Hashimoto thyroiditis, could be less uncommon than expected (7% of survivors) [95]. This could be attributed to the concept that infectious diseases and thyroid autoimmunity are related strictly in some predisposed patients. The main mechanisms involved in this relationship are attributable to molecular mimicry, polyclonal T cell activation by specific pathogen-related epitopes, and enhanced self-antigen presentation from thyrocytes (enhanced thyroid expression of human leukocyte antigen molecules) [96]. Nevertheless, data from SARS-CoV-2 are too inconsistent to bring conclusive assumptions, but it should be considered that autoimmunity may arise after a long-term period after infection. Therefore, it is not possible to confirm or exclude whether the SARS-CoV-2 pandemic could be affecting the incidence of autoimmune thyroiditis.

TSH seems to activate adipocytes to synthesize and secrete interleukin-6 (IL-6). Therefore, high circulating TSH levels, as observed in hypothyroid patients, may play a role in generating background systemic inflammation [97,98,99]. Moreover, thyroid hormone receptor agonism suppresses the macrophage release of IL-6 during experimental endotoxemia [100]. Considering this relationship, patients with uncontrolled hypothyroidism may be more susceptible to poor prognosis in the case of COVID-19. To explore this background gap, a cross-sectional analysis from the United States did not find any association between pre-existing hypothyroidism (composite of a previous diagnosis of hypothyroidism and levothyroxine prescription) and worse progression of COVID-19 in a whole cohort of patients (in- and outpatients) [101]. However, this analysis did not explore the results according to the baseline thyroid hormone balance [101]. More recently, another study found that levothyroxine supplementation and antithyroid medications for treating a pre-existing hypo- and hyperthyroidism, respectively, did not affect the prognosis of COVID-19 [102].

On the other hand, lower TSH and free (f) triiodothyronine (T3) levels have been observed in COVID-19 patients with poor prognosis in the context of the so-called euthyroid sick syndrome or non-thyroidal illness syndrome (NTIS). The NTIS is mainly attributable to systemic inflammation [103]. Elevated concentrations of circulating cytokines, such as IL-6, are involved in the pathogenesis of the syndrome [104]. In fact, the NTIS is observed in septic patients and could be associated with worse outcomes [105], as similarly in myocardial infarction [106] and acute heart failure [107,108]. T3 is essential to adapt injured tissue to hypoxemia [109] and reduce macrophage responsiveness to IL-6, suggesting a potential role of this hormone in contrasting systemic inflammation and exaggerated innate immune response [100]. In addition, in a randomized placebo-controlled trial, a 3-day consecutive infusion of T3 improved the left ventricle performance after myocardial infarction in patients with ventricular dysfunction and NTIS without any relevant adverse events [110]. In critically ill COVID-19 patients, serum fT3 was reduced and independently associated with all-cause mortality [111]. More recently, the NTIS on admission was found to predict clinical deterioration also in mild or moderate COVID-19 cases irrespective to SARS-CoV-2 viral load, age, and biomarkers of inflammation and tissue damage [112]. Hence, the clinical matter is whether the NTIS may be only a biomarker or, rather, it could serve as a risk factor for poor prognosis in septic patients [113,114,115]. The results of phase II, multicenter, prospective, randomized, double-blind, placebo-controlled trials in COVID-19 patients will provide a possible answer to this question [116].

IL-6 might induce the onset or relapse of hyperthyroidism in patients with Graves’ disease (GD) [95], and GD relapse cases have been described in patients after the diagnosis of COVID-19 [93,117]. It is hard to establish precisely the relationship between the two conditions. From a pathophysiological point of view, IL-6 may stimulate TSH receptor expression, as demonstrated in human orbital preadipocyte fibroblast, and high levels of IL-6 may be a potential trigger of inflammation in GD [118]. Insulin-like growth factor 1 plays a role in the pathogenesis of Graves’ ophthalmopathy, as similarly described in severe SARS-CoV-2-related lung injury, and its receptor may be a common pathophysiological target for both these conditions [119,120]. No data currently available let the authors suppose that GD, per se, might increase the risk of contracting or transmitting SARS-CoV-2 infection. However, hyperthyroidism could foster a worse clinical progression, a more prolonged hospital stay, and a higher in-hospital mortality rate [94]. This burden is related to possible detrimental consequences of untreated hyperthyroidism at the cardiovascular system and coagulative level [121,122]. Besides, hyperthyroidism may increase the susceptibility to worse progression in case of infection by reducing the immune system efficicency [123]. When a clinical suspicion of hyperthyroidism is relevant, accurate laboratory and instrumental workup are required to confirm or rule out the condition, and adequate treatment is necessary to restore euthyroidism. Antithyroid drugs (ATD) are the first-line therapy of hyperthyroidism. It should be noted that ATD infrequently induce neutropenia or agranulocytosis, therefore, predisposing to infectious diseases, including those involving the respiratory tract [124,125]. Initial symptoms of SARS-CoV-2 infection, such as fever or pharyngodynia, may be similar to those observed in patients with airways infection due to thionamide-induced agranulocytosis. A prompt white blood count should be obtained in this setting to discriminate the two clinical conditions (e.g., neutropenia vs. lymphocytopenia) [126]. Of note, thionamide-induced agranulocytosis is usually a dose-dependent adverse event, especially for methimazole, while it is unpredictable with propylthiouracil [127]. A cross reaction among thionamides is also well known; therefore, the shift from methimazole to propylthiouracil or vice versa is not recommended and the patient should be managed alternatively [127].

It has not been well recognized if thyroid carcinomas should be considered risk factors for poor prognosis in COVID-19. Of note, primary thyroid malignancies are mostly well-differentiated papillary carcinomas that often have a favorable long-term prognosis. However, diagnostic workup of thyroid malignancies has been affected as the consequence of pandemic-related restriction. An Italian experience showed that the number of thyroid fine-needle aspiration procedures was considerably restricted during the ongoing pandemic [128]. These data are consistent with the global scenario. Even though thyroid nodules are malignant lesions, rarely a delayed cytological assessment of highly suspicious nodules may lead to potentially relevant clinical consequences, especially for malignant nodules with malignant cytology [129]. In a pandemic scenario, a careful, pre-operative, nodule risk stratification is even more essential to better deal with this matter. Therefore, patients with thyroid nodules at high risk of malignancy should undergo cytology without relevant delays, whereas patients with moderate- or low-risk nodules should be safely postponed for any cytological procedures, waiting for better epidemiological conditions [29]. Similarly, patients who require endocrine surgery should be prioritized for undelayable interventions [130,131]. The management of these patients should be scheduled considering the local rate of COVID-19 transmission, hospital resources, and availability of intensive care units [129]. Accordingly, patients with thyroid tumors requiring acute airway management should undergo surgery as soon as possible. The treatment of low-risk papillary carcinomas and benign conditions may be postponed safely in the vast majority of the cases [132,133].

Radioactive iodine therapy should be considered an alternative to surgery or medications in case of adverse events. It is usually indicated for the completion of thyroidectomy in patients with high-risk differentiated thyroid carcinomas [134], recurrence of hyperthyroidism in GD [135], or in case of autonomously functioning thyroid nodules [136]. As observed for other healthcare services, the choice of nuclear medicine has been restricted since the pandemic occurred [137]. However, since radioiodine has only mild effects on the immune system, it can be administered to patients safely, organizing the sessions according to clinical priorities [137].

## 5. Parathyroid Glands and Calcium-Phosphorus Metabolism

Calcium might play a role in COVID-19 infection since it seems to be involved in endosome trafficking and viral internalization into host cells and the process of viral fusion to the cell membrane of many enveloped viruses, including coronaviruses [138].

Vitamin D (VD) and its active metabolites are essential for maintaining homeostatic balance in several tissues and systems, such as bone, skin, heart and vessels, pancreas, muscles, and brain, and, last but not least, carries immune-modulative actions [139,140]. Sunlight exposure, particularly ultraviolet B radiation, is necessary to activate VD precursor (7-dehydrocolesterol) in the skin. However, VD is also taken by food intake from both animal and vegetable sources [141]. The leading cause of VD deficiency is inadequate exposure to sunlight, which is not replaced or enforced by a sufficient VD intake through nutrition. However, other factors affect skin VD activation or bioavailability, including sunscreens, dark skin color, intestinal malabsorption, and overweight/obesity [142]. To date, VD deficiency (VDD) is a common finding among the general population worldwide [143], even if it is largely influenced by feeding behaviors (e.g., fortified food consumption is high in Northern Europe) [144]. VD may modulate immune system function, as observed in autoimmune thyroid diseases [145,146]. Activated antigen-presenting cells (APCs), natural killer (NK), T-cell CD4+, and B-cells typically express VD receptors on the cell surface and VD may modulate their activity [147]. Specifically, the active form of VD calcitriol (1,25(OH)2-VD) directly inhibits T helper 1 and 17 development and enhances T helper 2 and regulatory differentiation T-cells [148,149]. Macrophages express the enzyme 1 alpha-hydroxylase and may synthesize the 1,25(OH)2-VD. The 1 alpha-hydroxylase and VDR synthesis are both enhanced by interferon-gamma and Toll-like receptors 2/1 activation. Thus, the levels of 1,25(OH)2-VD increase considerably during inflammation [150]. On the other hand, 1,25(OH)2-VD suppresses the synthesis of the Toll-like receptors, generating negative feedback on the innate immune response. VD enhances the expression of cathelicidin and beta-defensin 4 that potentiates the macrophage response against pathogens [147,151]. Additionally, 1,25(OH)2-VD suppresses the expression of major histocompatibility complex class II and co-stimulatory molecules, thus blunting the APCs’ presenting-cell activity [152]. Additionally, calcitriol may suppress the synthesis of IL-17 by a post-transcriptional mechanism in T-helper 17 [153], increase T-cells’ sensibility to extrinsic death signals [154], and increase the synthesis and release of IL-10 from B-cells [155]. VDD (<20 ng/dL) was found to increase the risk of respiratory infections, fostering the progression of pulmonary diseases, and was related to poor prognosis in patients admitted to intensive care units [156]. Additionally, VD supplementation has a role in protecting against respiratory tract infections [157]. It can antagonize the IL-6 pathway and suppress the pro-inflammatory cytokine response of macrophages and respiratory epithelial cells to different types of viruses [158,159]. The fatality rate for respiratory infection is high in some geographical areas with a higher prevalence of VDD, especially during cold seasons, and VD replacement may improve poor outcomes in patients with severe VDD [160].

As previously shown with other coronaviruses’ infection, such as SARS [161], Jia-Kui Sun et al. retrospectively found that patients with hypocalcemia (total serum calcium <8 mg/dL) at admission had worse clinical and laboratory parameters, higher incidence of organ failure and septic shock, and higher 28-day mortality [162]. This trend was also similar when correlations were assessed by using the levels of 25-hydroxide VD [162]. However, as a limitation, the analyses were performed only considering total serum calcium concentration, not ionized or adjusted calcium serum levels. A high prevalence of hypocalcemia in patients admitted to hospital was observed in a retrospective study including 531 patients with COVID-19. Hypocalcemia was found more frequently in elderly males, resulting in an independent risk factor associated with hospital admission in multivariate analyses and intensive care unit admission and mortality in univariate analysis [163]. These data are consistent with other findings reported in critically ill individuals. At admission and over 4 days after the hospitalization, severe hypocalcemia rather than mild hypocalcemia or normal calcium levels was associated with poor prognosis and greater mortality in critical illness [164]. In a systematic review and meta-analysis, hypocalcemia was related to poor prognosis, and the mean difference in serum calcium levels among patients with poor outcomes (26%) was 0.69 mg/dL [165]. Given the importance of calcium-phosphorus metabolism in this clinical scenario, Bennouar et al. found that a cutoff value of 16 ng/mL for VD and 8.2 mg/dL for corrected calcium could predict poor prognosis (hazard ratios 6.9 and 6.2, respectively) in COVID-19 patients with a sensitivity of 76% and 84% and a specificity of 69% and 60%, respectively [166].

Baseline VD status seems not to influence the risk of contracting SARS-CoV-2 infection, as recently published from Brazil [167]. In other words, a sufficient VD status may fail to protect against SARS-CoV-2 infection. However, VDD may negatively affect the prognosis once the infection has occurred. As indirect proof, patients living below rather than above the 35° latitude exhibited a lower mortality rate due to COVID-19 and this result could be possibly related with a lower prevalence of VDD among the general population [168]. More precisely, the prevalence of VDD among hospitalized COVID-19 patients was higher than the general population [169], especially in those with more severe acute respiratory failure and higher mortality risk [170]. VD supplementation for improving the prognosis in COVID-19 patients has a pathophysiological rationale, but its efficacy should be further investigated [160,171]. From this point of view, VDD may be a predisposing factor to COVID-19 infection and aggressiveness, but observational studies are needed to explore the role of VDD among hospitalized patients. For instance, data from 391 severe COVID-19 patients confirmed the formulated hypothesis, even if some confounding factors, such as the type of measured VD metabolites, assay methods, age, concomitant comorbidity, and nutritional status, may have affected the results partially [172]. Therefore, the role of VD supplementation in COVID-19 patients awaits the results of well-designed experimental studies (NCT04535791 [173]; NCT04366908 [174]).

VD supplementation remains indicated according to guidelines, especially in elderly and comorbid people in home confinement [175]. Of note, COVID-19 severity and mortality are high in the elderly and patients with diabetes mellitus, chronic lung, cardiovascular and renal diseases, and malignancies [15,176]. Furthermore, obesity, especially when the body mass index is over 35 Kg/m2, is another risk factor for COVID-19 worse progression and mortality [177,178]. Several mechanisms have been proposed for explaining this excess of risk, including a relevant background systemic inflammation, hormone imbalance (especially in men) [179], cardiometabolic diseases (including diabetes mellitus) [180], and reduced respiratory reserve [179,180]. In addition, VDD is more frequent and severe in obese patients due to lifestyle habits (e.g., limited exposure to sunlight) and VD sequestration in adipose tissue [181]. The pandemic-related lifestyle changes, including reduced time spent outdoors and persistent home confinement or immobilization in case of extended hospital stay due to COVID-19, are other risk factors for VDD. Therefore, its supplementation is necessary to improve musculoskeletal outcomes and prevent falls, frailty, and fracture in these at-risk categories [156].

The abovementioned results suggest a possible role of serum calcium, particularly the ionized form, and VD as valuable biomarkers of disease aggressiveness. Therefore, patients with pre-existing hypocalcemia should be advised to well recognize the signs and related symptoms of hypocalcemia and, in this case, to take additional calcium tablets (500 to 1000 mg per day as an add-on to current treatment) or take calcium-rich or calcium-fortified foods or beverages [182]. However, blood samples to confirm hypocalcemia and medical consultation are required as soon as possible for appropriate therapy adjustments. In case of vomiting or diarrhea, intestinal calcium absorption may be reduced. If total serum calcium < 7.5 mg/dL, calcium supplementation should be administered parenterally as gluconate calcium 10% one to two vials, corresponding to around 90–180 mg of elemental calcium [182]. Magnesium supplementation is necessary when hypomagnesemia is a concomitant, rather than the leading, cause of hypocalcemia. 

The management of calcium-phosphorus imbalances may be revised during the ongoing pandemic. Parathyroidectomy for treating new-onset primary hyperparathyroidism should be delayed, as it usually evolves slowly over time. Mild hypercalcemia (<12 mg/dL) may be monitored over time, while medical treatment is required for moderate or symptomatic hypercalcemia (>12 mg/dL), and calcimimetics may be considered before the definitive selective parathyroidectomy [183].

In case of confirmed severe acute hypercalcemia manifesting with nausea or vomiting, anorexia, confusion, arrhythmias, and polyuria, saline solution 0.9% is usually effective in reducing serum calcium levels and restore the hydric imbalance. Fluids may be administered rapidly once the diagnosis has been made (500–1000 mL) and continued at the rate of 200–250 mL/h. Loop diuretics may be administered to enhance calciuresis, especially in elderly patients or those with concomitant chronic or decompensated cardiovascular and renal diseases, to avoid possible fluid overload [184]. New-onset hypercalcemia necessitates an accurate diagnostic workup. In most cases, hypercalcemia is attributable to primary hyperparathyroidism and malignancies, while other causes include thiazides, thyrotoxicosis, adrenal insufficiency, hypervitaminosis D, and familial hypocalciuric hypercalcemia [184]. Non-parathyroid hypercalcemia, such as malignant hypercalcemia, may require bisphosphonate treatment, such as zoledronic acid, while Denosumab, a fully humanized monoclonal antibody targeting the receptor activator of nuclear factor-kB ligand (RANKL), is another therapeutic strategy for controlling hypercalcemia [183,185].

## 6. Primary Osteoporosis

Primary osteoporosis and fragility fractures management can pose a real challenge for an appropriate diagnosis and management during the COVID-19 pandemic. Dual X-rays absorption (DXA) scan is the gold standard diagnostic tool for osteoporosis. However, DXA scan remains an elective procedure and its use has been usually restricted since the pandemic occurred. The clinical decision to treat may be based on an accurate anamnestic collection and prediction tools for long-term osteoporosis fracture risk [186], while DXA scanning can be postponed according to local epidemiological conditions [186]. VD and calcium supplementation, along with oral bisphosphonates and healthy lifestyle behaviors, remain the first-line therapy for the primary prevention of fragility fracture in postmenopausal osteoporosis [187]. Therapy can be continued, as these agents demonstrated long-term efficacy and safety [188]. For patients receiving teriparatide, denosumab, and zoledronic acid, some aspects should be considered. Patients taking denosumab should continue the treatment twice a year along with VD and calcium supplementations to prevent possible hypocalcemia. Considering that denosumab has a rapid “on/off” effect, the scheduled administration should not be interrupted abruptly or indefinitely delayed [183], as the bone mass loss and fracture risk increase significantly in a few months, especially in the case of considerable delays [189]. Patients should also be monitored at a distance, especially for laboratory exams, while those obtaining adequate control may be rescheduled for delayable follow-up visits, and prescriptions should be renewed automatically. If denosumab is discontinued, alternative bisphosphonates should be prescribed to attenuate the consequent bone demineralization [190]. Compared to bisphosphonates, denosumab did not require dose adjustment in renal insufficiency; however, patients with a pre-existing or occurring renal impairment with a glomerular filtration rate less than 30 mL/min may have a greater risk of hypocalcemia. The RANK-RANKL signaling may modulate immune functions, as it is involved in the self-tolerance selection of lymphocytes in the thymus and may modulate lymph node maturation, presenting-cell activity, and immunosuppression [191]. Despite initial concerns related to an increased risk of severe infections among patients on denosumab than placebo [192], there is no current evidence that denosumab may increase this risk significantly [193].

Zoledronic acid at 5 mg once yearly may be considered as an alternative in patients who did not tolerate oral bisphosphonates or with gastrointestinal complaints or malabsorption [194]. Zoledronic acid should be administered intravenously over at least 30 min to avoid acute reactions. Therefore, this procedure is frequently carried out in healthcare facilities and may be susceptible to be restricted or delayed during the pandemic. Anti-resorptive zoledronic acid action persists after discontinuation for months, showing a more favorable profile compared to denosumab in interrupted or delayed administration. As suggested by expert opinions, zoledronic acid can be delayed for 6 to 9 months without any relevant rebound on bone mass loss and fracture risk [183].

Teriparatide, a recombinant human parathyroid hormone (1–34), has a rationale for contrasting the pathophysiological mechanism of osteoporosis in primary postmenopausal osteoporosis, glucocorticoid-induced secondary osteoporosis, and osteoporosis in hypogonadal men [195]. It is generally administered once a day at 20 mcg continually over 24 months, but other regimens have demonstrated similar efficacy [187]. Bone mass loss is fast when discontinuing anabolic agents, even if it is not rapidly associated with a relevant short-term rebound in the fracture risk [183,196]. Patients should be advised to avoid unnecessary discontinuation of teriparatide, and prescriptions should be renewed automatically even in the case of deferred follow-up visits. If anabolic agent continuation is not feasible, antiresorptive agents, such as oral bisphosphonates, may be prescribed until a newer clinical reassessment [186].

The reduced time spent outdoors seems to have a biphasic effect on fracture epidemiology. On the one hand, the incidence rate of traumatic fractures dropped due to reduced exposure to possible injuries related to work and outdoor activities (e.g., sports, travels). On the other side, home confinement, loss of mobility, and reduced exposure to sunlight may have potentially detrimental consequences on frailty fracture. Then, adequate measures of bone loss and fracture preventions should be implemented during the pandemic, including pharmacological and non-pharmacological approaches in order to reduce possible burdens. Moreover, healthcare constraints may complicate the surgical management of fractures, which may affect mortality, especially for elderly and severely affected osteoporotic patients [197]. Lastly, it should be considered that COVID-19 patients may experience bone mass loss directly due to systemic inflammation, cytokine syndrome, prolonged home confinement, immobilization during the hospital stay, and possibly glucocorticoid treatment [198]. The final effect on bone health and fracture risk is currently unknown, but it is reasonable to consider this cluster of patients as at higher risk, mainly elderly and comorbid people.

## 7. Adrenal Gland

Different endocrine societies produced recommendations for patients with adrenal insufficiency or endogenous hypercortisolism [199,200]. Glucocorticoids play a pivotal role in priming the immune system as the consequence of infection or injuries. Hypothalamic-hypophysis activity also increases suppression of the immune response and reduces cytotoxic damage and possible autoimmune responses. According to this interesting point of view, patients with primary or secondary hypocortisolism are at increased risk of poor prognosis if glucocorticoid replacement is not adequately supported during the different phases of the infection, including COVID-19 [201]. This is in accordance with other findings reporting a higher frequency of lower respiratory tract infection in patients with primary adrenal insufficiency (PAI) [202]. More specifically, patients with PAI display a significant mortality excess than the general population, which is at least doubled in men and tripled in women [203]. Deaths are commonly due to acute adrenal insufficiency, and infectious diseases may significantly increase this risk, especially for younger patients and more considerably in men than women [204].

One pivotal study found that NK cell cytotoxicity, which has an essential role in antiviral immune defense, was impaired in PAI patients [205]. Moreover, a blunted response of peripheral blood mononuclear cells to interferons’ stimulation was observed, too [206]. On the other hand, it is also known that acute infectious diseases may induce bilateral adrenal bleeding or adrenal venous thrombosis, leading to acute adrenal insufficiency and mortality excess in fatal sepsis [207]. In addition, adrenal insufficiency may result from a direct or immune-mediated adrenal injury after different acute infectious diseases, mostly with viral etiology [208]. For instance, autoptic studies in SARS deceased patients had shown degeneration and necrosis of adrenal cortical cells, and SARS-CoV was identified in the adrenal glands, possibly confirming a direct cytopathic effect [209]. As ACE2 is widely expressed at adrenal levels, it is believed that SARS-CoV-2 may lead to similar detrimental consequences as described for other coronaviruses [209], even if a causal relationship between new-onset PAI and SARS-CoV-2 remains to be confirmed [210,211]. Microscopic examination of the adrenal glands from COVID-19 deceased patients showed acute fibrinoid necrosis of small vessels, mainly arterioles, in adrenal parenchyma, capsule, and periadrenal adipose tissue with subendothelial vacuolization and apoptotic debris without significant signs of inflammation, parenchymal infarctions, or thrombosis [212]. Venous thromboembolism and coagulopathy are frequently occurring complications in COVID-19 patients, especially in severe cases [213]. Thus, it could not be excluded that an acute adrenal injury may occur after venous thromboembolism or bilateral adrenal hemorrhage in some cases [214].

Cortisol dynamics may be altered in patients with coronavirus infections, even if the literature is insufficient in this regard. Interestingly, some immunogenic epitopes expressed on coronavirus surfaces display a significant homology level with the mammalian ACTH. Consequently, some antibodies directed against the viral envelope may potentially destroy ACTH-secreting cells, thereby affecting the hypothalamic-pituitary-adrenal axis [37]. This hypothesis remains to be confirmed, even if a careful study of the hypothalamic-pituitary-adrenal axis is difficult, especially among severely ill patients who are mostly on glucocorticoid treatment.

Lymphopenia is a common finding in patients with COVID-19 and is related to poor prognosis [215]. The pathogenesis of lymphopenia in this clinical setting depends on different factors, such as higher systemic inflammation and cytokine storm (IL-6), T-cell exhaustion, excess of programmed cell death, and SARS-CoV-2 direct T-cells’ affection [216]. In addition, as observed in a previous experience with SARS patients, peripheral lymphocyte count was inversely correlated to serum cortisol levels and the contrary was observed with neutrophils [217]. As an acute adrenal crisis is the leading cause of death in infected patients with a pre-existing adrenal insufficiency [218,219], a prompt recognition of signs and symptoms suggestive for glucocorticoid insufficiency and adequate glucocorticoid replacement are mandatory [34,90]. In this setting, lymphocyte cell count may be a less reliable biomarker of adrenal insufficiency in COVID-19 patients. Therefore, the suspicion should be recognized based on hemodynamic and sera electrolytes if available. Asymptomatic patients who tested positive for COVID-19 do not require any glucocorticoid supplementation unless signs and symptoms of systemic infection occur. In case of fever, oral glucocorticoid supplementation is necessary, and the total daily dose should immediately be doubled or tripled, up to 20 mg hydrocortisone every 6 h [78]. When clinical conditions deteriorate by vomiting or diarrhea, the patient should be referred to an emergency department urgently, and hydrocortisone should be administered already at home along with resuscitation fluids intravenously if feasible. After the first parenteral dose (100 mg), another 200 mg of hydrocortisone are usually recommended over the 24 h, continuously or intermittently [78]. In PAI, fludrocortisone replacement should be continued at the habitual dose in asymptomatic or mild cases. However, when hydrocortisone replacement has been potentiated (>50 mg/day), it should be discontinued to avoid excessive sodium retention and kaliuresis. Fludrocortisone should be re-started at the habitual dose when the total daily dose of hydrocortisone has tapered under 50 mg per day [78].

Patients with PAI should be educated about hygienic tips to observe during the pandemic in public and at work or school [78]. In those with stable diseases and without COVID-19 complaints, follow-up should be continued regularly also, and telemedicine may be a tool for improving the management at distance.

As observed in uncontrolled Cushing syndrome (CS), hypercortisolism may increase the cardio-metabolic risk [220,221] and induce thrombosis [222], immunosuppression, and background systemic inflammation [223]. This consideration is also proper in patients with exogenous overexposure to glucocorticoid therapy. In patients with CS, cortisol normalization is one of the most important therapeutic targets, but it is usually challenging to be achieved and some of the affected patients remain uncontrolled despite non-surgical and surgical management [224,225]. As patients with CS may be at higher risk of poor prognosis once an infection occurs, including COVID-19, they should be advised to maintain adequate behavior and observe hygienic tips during the pandemic [226]. Cortisol excess and its related comorbidities, such as diabetes and hypertension, should be managed as best as possible, but in-person visits should be minimized by scheduling only patients requiring frequent imaging and laboratory investigations. It should be noted that respiratory tract symptoms may be the consequence of opportunistic pneumonia, especially in patients who did not achieve a prompt remission of glucocorticoid excess. In this case, patients should be referred to hospital care for receiving an adequate differential diagnosis. To avoid this burden, prophylaxis for opportunistic infection with trimethoprim/sulfamethoxazole may be of help [226].

From a diagnostic point of view, more emphasis must be given to the crucial clinical hallmarks of CS to investigate only cases in which a cortisol excess diagnosis would be more likely [227]. In questionable cases, the investigation should be deferred according to a “wait and see” approach. Patients with a moderate-to-severe clinical disease must be investigated and managed urgently to avoid a poorer prognosis [226]. Surgical or medical treatment are required to control CS and a 24-h urinary free cortisol measurement and serum morning cortisol are the best biomarkers of disease control. When CS has been confirmed and the etiology well established, surgery should be considered only for severe cases, whereas medical management may be more appropriate for the others [228]. Steroidogenesis inhibitors, such as ketoconazole, are the first-line therapy for CS. As ketoconazole might induce acute liver toxicity, liver function tests should be carried out monthly for the first 3–6 months after the treatment initiation or following any dose adjustments [229]. Glucocorticoid receptor antagonists, such as mifepristone, may be difficult to titrate and the prescription should be reserved in patients requiring rapid control of glucocorticoid-related comorbidities (e.g., uncontrolled hypertension or hyperglycemia, psychotic symptoms). As the risk of acute hypoadrenalism may be a clinical matter, especially in newly diagnosed patients, a block-and-replace regimen may be useful to adequately control the disease, reducing at the same time the requirement of recurrent medical consultations. Low-molecular-weight heparin should be considered in patients with higher cortisol levels until a better therapeutic target has been achieved [226].

Medical management of CS should be implemented in patients with COVID-19, as the risk of worse progression is considerable and some specific risk factors and clinical suggestions should be kept in mind when COVID-19 occurs in CS [230]. As some examples, prolonged duration of viral infectious disease and susceptibility to bacterial and fungal infections have been described in CS. Therefore, CS patients with COVID-19 could require antibiotics and antimycotic coverage for a prolonged time. Moreover, CS impairs glucose and arterial pressure control and patients with CS are at higher risk for thrombosis and thromboembolism. These alterations are likely to worsen the prognosis in COVID-19 patients and require a careful medical management (i.e., glucose-lowering agents, antihypertensive medication, and antithrombosis prophylaxis) [230].

## 8. Gonads

Despite heterogenicity, men, compared to women, should be considered more susceptible to SARS-CoV-2 infection, more severe disease, and worse prognosis [231]. The basis of this gender dimorphism could be related to baseline health status (i.e., chronic comorbidities), lifestyle (i.e., cigarette smoking), and hormonal and immunologic variables [179].

As another issue, biochemical and immunological factors may induce SARS-CoV-2 testicular damage through direct or indirect mechanisms, leading to possible worries for both endocrine and secretive function [232,233]. Two Chinese observational studies assessed the possible presence of SARS-CoV-2 in the semen of a few patients and found controversial results [234,235]. In another observational trial, crypto-azoospermia and high IL-8 levels were found in a relevant percentage of men (25%) who had recovered from COVID-19 and exceeded the frequency observed in the general population [236]. In a more extensive observational study enrolling 74 men with moderate-to-severe forms of COVID-19 (mean age 31 years), SARS-CoV-2 RNA was undetected in the semen and urogenital secretions, but a decline in sperm count was found in those specimens collected from patients with more than 90 days since recovery from the infection [237]. Best et al. compared the median of sperm count between men who tested positive for SARS-CoV-2 (sperm collection after positive real-time PCR testing; 11–64 days) and those negative. The median value of total sperm count was lower in SARS-CoV-2-positive than -negative men (12.5 million vs. 59.2 million, p < 0.002) even if this difference was attenuated when another seminal analysis was performed after 3 months from recovery of SARS-CoV-2 (only in five patients) [238]. Other observational studies, case series, and case reports found no evidence of SARS-CoV-2 RNA in sperm specimens, even in the presence of serum testosterone levels, sperm count decline, or both in acute and recovery phases [239,240,241,242,243].

It should be noted that data have been collected from a restricted number of cases and studies could be underpowered for specific purposes. Biases are also attributable to the nature of the studies, the lack of controls (negative patients or baseline seminal characteristics according to the studies’ designs), and possible confounding factors, such as baseline heterogeneity of participants’ characteristics (i.e., age, comorbidity, lifestyle) and time of sperm specimen collection [244]. Other hypotheses may be considered for explaining the results, especially in interpreting quantitative and qualitative sperm changes, including the effect of fever and considering the severity of clinical presentations (semen collection was usually carried out within 3 months since the diagnosis of SARS-CoV-2 infections), medications assumed for treating COVID-19 (i.e., antibiotics, corticosteroids), and sexual abstinence [245].

Since the first hypothesis, T was identified as a critical hormone in COVID-19 with a possible bivalent effect [233,246]. On the one hand, normal serum T levels may foster a grand viral entry into host cells and facilitate systemic dissemination of SARS-CoV-2. On the other side, lower T levels, as observed in aged and comorbid men, may predispose them to poor prognosis due to a possible role of male hypogonadism in inducing cardiovascular events, exaggerating immune system and coagulative response [179,247]. These assumptions have been partially supported by observational pilot studies that found lower serum levels of T in COVID-19 men exhibiting poor prognosis after hospital admission, possibly in a context of primary testicular damage [248,249,250]. Given that, serum T concentrations may be a biomarker of poor prognosis in hospitalized patients and could be helpful to screen precociously those patients who require more intensive treatment. Additionally, T replacement therapy should be continued in patients with male hypogonadism, according to the general recommendations [251], and caution should be used in introducing therapies aiming to reduce T or increase estradiol serum levels in men with confirmed COVID-19 until specific clinical trials are carried out.

Several observations from different countries have revealed that the frequency of worse outcomes was lower in women than men [252,253]. The basis for a different prognosis in COVID-19 may be attributable to malfunctioning immune system response. An exaggerated innate immune response has been well recognized in COVID-19 patients exhibiting poor prognosis, and it could be related to a defective or delayed adaptive immune response intervention. It is characterized by a remarkable release of cytokines, such as IL-6, IL-1b, and tumor necrosis factor-a (TNF-a), and manifesting with lung infiltration, acute respiratory distress syndrome, and multiorgan failure [254,255].

The X chromosome contains several genes regulating the immune system response, and this biological condition may be responsible for a gender dimorphic immune system regulation [256,257,258].

Estrogens and progesterone (P4) could be protective against a worse progression of COVID-19 [259]. Indeed, 17β-estradiol (E2) and P4 showed a significant immunomodulatory action. Both estrogenic receptors (ERa and ERb) are found in peripheral blood mononuclear cells, with ERa being more evident in CD4+ T lymphocytes, while B cells express higher levels of ERb. Lower but similar ERs’ levels are expressed in blood CD8+ T lymphocytes and monocytes [260]. High physiological E2 concentrations can affect both macrophages and B cells. In particular, E2 suppresses macrophages’ production of pro-inflammatory cytokines (IL-6, IL-1β, and TNF-α), and the chemokine CCL2, which prevents neutrophils’ and monocytes’ migration into inflamed tissue. E2 stimulates antibody production from B cells, and progesterone also inhibits pro-inflammatory IL-1β and IL-12 productions by macrophages and dendritic cells. Finally, E2 and P4 improve regulatory T cells (Treg), facilitating immune tolerance. For instance, pregnancy is a biological model of anti-inflammatory status aiming to avoiding fetal rejection (immune tolerance) and favoring the passive transfer of maternal antibodies to the fetus [261]. High levels of E2 during pregnancy may enhance antibody production by B cells [262].

Pregnant women are not entirely protected from COVID-19 but could be affected by a usually more favorable clinical presentation [263]. A retrospective Chinse study in 118 pregnant women with COVID-19 pneumonia reported only nine cases (8%) of severe pneumonia with hypoxemia. Notably, in six out of nine women, the exacerbation of pneumonia occurred during the postpartum period when serum E2 and P4 went down steeply [264]. Pregnant women with COVID-19 display an increased risk of preterm delivery and cesarean section. No evidence suggests a possible vertical transmission of SARS-CoV-2 infection, but a placental transfer of neutralizing antibodies from infected mothers to children has been described [265,266]. Evidence of SARS-CoV-2 on female fertility is an issue, and specific research will clarify the issue [267].

ACE2 gene is located to the X chromosome, in an area (Xp22) where genes are reported to escape from X-inactivation. This condition leads to a greater expression of ACE2 enzyme on cell membranes in women than men. However, TMPRSS2 also plays a crucial role in facilitating SARS-CoV-2 entry into the host’s cell as the enzyme catalyzes SARS-CoV-2 spike protein proteolysis, allowing the consequent viral fusion with host cells’ membrane [29]. The absence of TMPRSS2 significantly reduces SARS-CoV-2 internalization and replication into human airway cells, as observed experimentally [268]. Testosterone (but not estrogens) enhances TMPRSS2 expression, predisposing men to much more tissue exposure to SARS-CoV-2 than women. Sexual hormones seem to play a crucial role in co-regulating the cardiovascular system, and E2 are considered a protective factor for cardiovascular health in women [269]. This evidence is reliable even in the case of COVID-19 infection. Individuals with cardiovascular diseases are prone to develop a severe form of COVID-19 infection, and women could be more protected than men thanks to a protective E2 effect, especially during the premenopausal phase. Endothelial injury repair is more efficient in women, and E2 accelerates collateral vessel formation in ischemic tissues [270]. The leading mechanisms encompass the synthesis and release of vasoactive substances, such as endothelial nitric oxide and prostacyclin, and antiplatelet agents and the inhibition of pro-inflammatory mediators following a vascular injury [271]. This protection is mainly observed in fertile women and is usually lost during the menopausal transition when circulating E2 levels decline remarkably [269]. Several preclinical and clinical studies support the protective role of E2 during viral infectious diseases, especially in earlier stages [272]. Bilateral ovariectomy or treatment with estrogen receptor antagonists, causing an acute estrogen deprivation, dramatically amplified the severity and mortality of SARS-CoV infection in female mice. Conversely, ovariectomized mice treated with tamoxifen, a selective estrogen receptor modulator, recovered from this risk, as observed in another coronavirus infection [273]. Similar data have been reported in animal models of influenza, in which estrogens’ therapy induced a more appropriate innate immune response in the lungs, with a reduction in pro-inflammatory cytokines and chemokine responses before the outbreak of the overt clinical disease [274]. In a mouse model of SARS-CoV infection, females, compared to males, had lower virus titers and circulating monocyte, macrophages’, neutrophils’, cytokines’ (IL-6, IL-1β, and TNF-α), and chemokines’ levels, fostering a better prognosis and lower mortality [275]. Additionally, regular estradiol hormone therapy has a positive effect on the survival rates in post-menopausal women [276].

The polycystic ovary syndrome is a systemic condition predisposing to a greater cardiometabolic risk than the general population [277]. In addition, as women with PCOS are exposed to hyperandrogenism and considering that androgens increase the expression of TMPRSS2, they could be exposed to a worse progression of COVID-19 once the infection has occurred [278]. According to the recently published observational retrospective study, women with PCOS had a 28% increased risk of contracting SARS-CoV-2 infection than those without PCOS and, consequently, the former should be encouraged to observe hygienic measure to avoid burden during the COVID-19 pandemic [279].

## 9. Summary and Perspectives

COVID-19 can lead to different matters in clinical endocrinology practice (Supplementary Table S1) First, possible endocrine derangements and hormonal imbalance may occur as new-onset symptoms or previous endocrine diseases’ recurrence. In these cases, it is necessary to well establish the timing and modality of symptom onset, especially in hospital settings. Additionally, it could be useful to exclude possible links between these clinical conditions with a recent SARS-CoV-2 infection to provide further scientific information about the topic. For this purpose, long-term follow-up could be useful to screen possible endocrine disorders in those patients who recovered from COVID-19 (e.g., autoimmune thyroiditis, male hypogonadism, erectile dysfunction).

Second, pre-existing endocrine and metabolic disorders and their related therapy may be risk factors for a greater risk of contracting SARS-CoV-2 infection or exhibiting worse clinical progression of COVID-19. No proofs support the fact that endocrine diseases may increase the risk of contracting or transmitting SARS-CoV-2, even if in some report women with PCOS appeared more susceptible to contract the infection. However, some of them are prone to develop a worse progression of COVID-19. For instance, patients assuming glucocorticoid replacement therapy for adrenal insufficiency or those with calcium-metabolism imbalance or uncontrolled endocrine diseases, such as hypercortisolism, may have higher risk of poor prognosis or death than the general population. As this risk could be related with the magnitude of the endocrine dysfunction, patients necessitate specific education to adequately manage chronic therapy and prevent the occurrence of SARS-CoV-2 infection. As these patients may be considered as at-risk population, possible endocrine imbalances should be promptly managed in case of an occurring SARS-CoV-2 infection in hospital as well as at home. Possible interactions between ongoing therapy for controlling endocrine disorders and contingent COVID-19 treatment should be also considered. This is the case of patients with hypercortisolism, among whom the risk of drug interactions could induce detrimental consequence, including hypoglycemia, QT prolongation, hypoglycemia, and hepatotoxicity.

Last, but not least, the management of endocrine diseases has been affected due to the ongoing pandemic. The phenomenon is still classified as a pandemic and some years could pass until the disease will become endemic and more manageable [280]. It is expected that long-term healthcare restrictions may hamper the management of endocrine diseases over time, leading to diagnostic delays and loss of disease control. The leading cause of this burden is largely attributable to impaired scheduling of follow-up visits. To better cope with the difficult situation, case triaging could be a valuable tool to prioritize patients requiring non-deferrable assessment for diagnostic or therapeutic purposes. Non-urgent elective appointments, laboratory and imaging assessments, and non-urgent surgery should be postponed safely. Remote consultations and digital telehealth solutions should be useful for patients and caregivers in less severe cases, or minor consultations or educational/informative purposes.

Finally, vaccines may be a resource in this historical context [281], despite the ongoing occurrence of novel and more transmissible SARS-CoV-2 variants that may lead to possible concerns [282]. Different types of vaccines are currently under investigation but some of them have received the approval for clinical use [283] as they demonstrated to be safe and effective in clinical trials, especially in reducing the risk of severe disease and death [284,285,286]. No data regarding vaccine safety and efficacy are currently available in specific clusters of at-risk patients, especially considering those with endocrine diseases. However, there are no reasonable concerns or real worries of thinking that vaccines may have a lower efficacy or safety uncertainties in patients with endocrine diseases. Therefore, they can follow the same rules as the general population according to the European Society of Endocrinology recommendation [287]. Patients with adrenal insufficiency could require supplemental glucocorticoid dosage with vaccine administration due to the occurrence of possible side effects (e.g., fever, myalgia, or arthralgia) [288]. Accordingly, patients with endocrine diseases should be included in the vaccination program, possibly with priority, especially considering patients with PAI, CD, CS, acromegaly, hypopituitarism, hypogonadal men, and polycystic ovary syndrome. The achievement of adequate vaccination coverage among these patients could reduce relevant clinical risks and allow safer access to healthcare facilities for diagnosis and cure.

## Data Availability

Data sharing not applicable.

## Supplementary Materials

The following are available online at https://www.mdpi.com/article/10.3390/jcm10132920/s1. Table S1. Summary of the main descriptive findings related to the three specific questions.

## Abbreviations

COVID-19	Coronavirus disease 2019
SARS-CoV	Severe Acute Respiratory Syndrome Coronavirus
SARS-CoV-2	Severe Acute Respiratory Syndrome Coronavirus 2
ACE2	Angiotensin-converting enzyme 2
TMPRSS2	Transmembrane protease serine 2
GH	Growth hormone
TSH	Thyroid-stimulating hormone
ACTH	Adrenocorticotropic hormone
CD	Cushing disease
NTIS	Non-thyroidal illness syndrome
GD	Graves’ disease
ATD	Antithyroid drugs
IL-6	Interleukin-6
VD	Vitamin D
VDD	Vitamin D deficiency
1,25(OH)2-VD	Calcitriol
RANK (L)	Receptor activator of nuclear factor-kB (ligand)
DXA	Dual X-rays Absorption
CS	Cushing syndrome
APC(s)	Activated antigen-presenting cell(s)
NK	Natural killer
TNF-a	Tumor necrosis factor-alpha

## References

[1] Zhou P., Yang X.L., Wang X.G., Hu B., Zhang L., Zhang W., Si H.R., Zhu Y., Li B., Huang C.L. (2020). A pneumonia outbreak associated with a new coronavirus of probable bat origin. Nature.

[2] Gorbalenya A.E., Baker S.C., Baric R.S., de Groot R.J., Drosten C., Gulyaeva A.A., Haagmans B.L., Lauber C., Leontovich A.M., Neuman B.W. (2020). The species Severe acute respiratory syndrome-related coronavirus: Classifying 2019-nCoV and naming it SARS-CoV-2. Nat. Microbiol..

[3] Wu F., Zhao S., Yu B., Chen Y.M., Wang W., Song Z.G., Hu Y., Tao Z.W., Tian J.H., Pei Y.Y. (2020). A new coronavirus associated with human respiratory disease in China. Nature.

[4] Lu R., Zhao X., Li J., Niu P., Yang B., Wu H., Wang W., Song H., Huang B., Zhu N. (2020). Genomic characterisation and epidemiology of 2019 novel coronavirus: Implications for virus origins and receptor binding. Lancet.

[5] Ji W., Wang W., Zhao X., Zai J., Li X. (2020). Cross-species transmission of the newly identified coronavirus 2019-nCoV. J. Med. Virol..

[6] Li X., Zai J., Wang X., Li Y. (2020). Potential of large “first generation” human-to-human transmission of 2019-nCoV. J. Med. Virol..

[7] Andersen K.G., Rambaut A., Lipkin W.I., Holmes E.C., Garry R.F. (2020). The proximal origin of SARS-CoV-2. Nat. Med..

[8] (2020). The Lancet Respiratory Medicine COVID-19 transmission—Up in the air. Lancet Respir. Med..

[9] Jones N.R., Qureshi Z.U., Temple R.J., Larwood J.P.J., Greenhalgh T., Bourouiba L. (2020). Two metres or one: What is the evidence for physical distancing in covid-19?. BMJ.

[10] He J., Guo Y., Mao R., Zhang J. (2021). Proportion of asymptomatic coronavirus disease 2019: A systematic review and meta-analysis. J. Med. Virol..

[11] Bhuiyan M.U., Stiboy E., Hassan M.Z., Chan M., Islam M.S., Haider N., Jaffe A., Homaira N. (2021). Epidemiology of COVID-19 infection in young children under five years: A systematic review and meta-analysis. Vaccine.

[12] Zhang X., Tan Y., Ling Y., Lu G., Liu F., Yi Z., Jia X., Wu M., Shi B., Xu S. (2020). Viral and host factors related to the clinical outcome of COVID-19. Nature.

[13] Slifka M.K., Gao L. (2020). Is presymptomatic spread a major contributor to COVID-19 transmission?. Nat. Med..

[14] Gandhi R.T., Lynch J.B., del Rio C. (2020). Mild or Moderate Covid-19. N. Engl. J. Med..

[15] Onder G., Rezza G., Brusaferro S. (2020). Case-Fatality Rate and Characteristics of Patients Dying in Relation to COVID-19 in Italy. JAMA J. Am. Med. Assoc..

[16] Clark C.E., McDonagh S.T.J., McManus R.J., Martin U. (2021). COVID-19 and hypertension: Risks and management. A scientific statement on behalf of the British and Irish Hypertension Society. J. Hum. Hypertens..

[17] Mohammad S., Aziz R., Al Mahri S., Malik S.S., Haji E., Khan A.H., Khatlani T.S., Bouchama A. (2021). Obesity and COVID-19: What makes obese host so vulnerable?. Immun. Ageing.

[18] de Siqueira J.V.V., Almeida L.G., Zica B.O., Brum I.B., Barceló A., de Siqueira Galil A.G. (2020). Impact of obesity on hospitalizations and mortality, due to COVID-19: A systematic review. Obes. Res. Clin. Pract..

[19] Apicella M., Campopiano M.C., Mantuano M., Mazoni L., Coppelli A., Del Prato S. (2020). COVID-19 in people with diabetes: Understanding the reasons for worse outcomes. Lancet Diabetes Endocrinol..

[20] ElGohary G.M., Hashmi S., Styczynski J., Kharfan-Dabaja M.A., Alblooshi R.M., de la Cámara R., Mohmed S., Alshaibani A., Cesaro S., Abd El-Aziz N. (2020). The risk and prognosis of COVID-19 infection in cancer patients: A systematic review and meta-analysis. Hematol. Oncol. Stem Cell Ther..

[21] Lee S.C., Son K.J., Han C.H., Park S.C., Jung J.Y. (2021). Impact of COPD on COVID-19 prognosis: A nationwide population-based study in South Korea. Sci. Rep..

[22] Yin T., Li Y., Ying Y., Luo Z. (2021). Prevalence of comorbidity in Chinese patients with COVID-19: Systematic review and meta-analysis of risk factors. BMC Infect. Dis..

[23] Pellicori P., Doolub G., Wong C.M., Lee K.S., Mangion K., Ahmad M., Berry C., Squire I., Lambiase P.D., Lyon A. (2021). COVID-19 and its cardiovascular effects: A systematic review of prevalence studies. Cochrane Database Syst. Rev..

[24] Zhou F., Yu T., Du R., Fan G., Liu Y., Liu Z., Xiang J., Wang Y., Song B., Gu X. (2020). Clinical course and risk factors for mortality of adult inpatients with COVID-19 in Wuhan, China: A retrospective cohort study. Lancet.

[25] Wang D., Hu B., Hu C., Zhu F., Liu X., Zhang J., Wang B., Xiang H., Cheng Z., Xiong Y. (2020). Clinical Characteristics of 138 Hospitalized Patients with 2019 Novel Coronavirus-Infected Pneumonia in Wuhan, China. JAMA J. Am. Med. Assoc..

[26] Grasselli G., Zangrillo A., Zanella A., Antonelli M., Cabrini L., Castelli A., Cereda D., Coluccello A., Foti G., Fumagalli R. (2020). Baseline Characteristics and Outcomes of 1591 Patients Infected With SARS-CoV-2 Admitted to ICUs of the Lombardy Region, Italy. JAMA.

[27] Shi S., Qin M., Shen B., Cai Y., Liu T., Yang F., Gong W., Liu X., Liang J., Zhao Q. (2020). Association of Cardiac Injury with Mortality in Hospitalized Patients with COVID-19 in Wuhan, China. JAMA Cardiol..

[28] Amenta E.M., Spallone A., Rodriguez-Barradas M.C., El Sahly H.M., Atmar R.L., Kulkarni P.A. (2020). Postacute COVID-19: An Overview and Approach to Classification. Open Forum Infect Dis..

[29] Hoffmann M., Kleine-Weber H., Schroeder S., Krüger N., Herrler T., Erichsen S., Schiergens T.S., Herrler G., Wu N.H., Nitsche A. (2020). SARS-CoV-2 Cell Entry Depends on ACE2 and TMPRSS2 and Is Blocked by a Clinically Proven Protease Inhibitor. Cell.

[30] Hui K.P.Y., Cheung M.C., Perera R.A.P.M., Ng K.C., Bui C.H.T., Ho J.C.W., Ng M.M.T., Kuok D.I.T., Shih K.C., Tsao S.W. (2020). Tropism, replication competence, and innate immune responses of the coronavirus SARS-CoV-2 in human respiratory tract and conjunctiva: An analysis in ex-vivo and in-vitro cultures. Lancet Respir. Med..

[31] Puelles V.G., Lütgehetmann M., Lindenmeyer M.T., Sperhake J.P., Wong M.N., Allweiss L., Chilla S., Heinemann A., Wanner N., Liu S. (2020). Multiorgan and Renal Tropism of SARS-CoV-2. N. Engl. J. Med..

[32] Puig-Domingo M., Marazuela M., Giustina A. (2020). COVID-19 and endocrine diseases. A statement from the European Society of Endocrinology. Endocrine.

[33] Lazartigues E., Qadir M.M.F., Mauvais-Jarvis F. (2020). Endocrine Significance of SARS-CoV-2’s Reliance on ACE2. Endocrinology.

[34] Pal R., Banerjee M. (2020). COVID-19 and the endocrine system: Exploring the unexplored. J. Endocrinol. Investig..

[35] Leow M.K.S., Kwek D.S.K., Ng A.W.K., Ong K.C., Kaw G.J.L., Lee L.S.U. (2005). Hypocortisolism in survivors of severe acute respiratory syndrome (SARS). Clin. Endocrinol..

[36] Wei L., Sun S., Zhang J., Zhu H., Xu Y., Ma Q., McNutt M.A., Korteweg C., Gu J. (2010). Endocrine cells of the adenohypophysis in severe acute respiratory syndrome (SARS). Biochem. Cell Biol..

[37] Pal R. (2020). COVID-19, hypothalamo-pituitary-adrenal axis and clinical implications. Endocrine.

[38] Wheatland R. (2004). Molecular mimicry of ACTH in SARS–Implications for corticosteroid treatment and prophylaxis. Med. Hypotheses.

[39] Therapeutic Management|COVID-19 Treatment Guidelines. https://www.covid19treatmentguidelines.nih.gov/therapeutic-management/.

[40] Horby P., Lim W.S., Emberson J.R., Mafham M., Bell J.L., Linsell L., Staplin N., Brightling C., Ustianowski A., RECOVERY Collaborative Group (2020). Dexamethasone in Hospitalized Patients with Covid-19—Preliminary Report. N. Engl. J. Med..

[41] Sterne J.A.C., Murthy S., Diaz J.V., Slutsky A.S., Villar J., Angus D.C., Annane D., Azevedo L.C.P., Berwanger O., Cavalcanti A.B. (2020). Association between Administration of Systemic Corticosteroids and Mortality among Critically Ill Patients with COVID-19: A Meta-analysis. JAMA J. Am. Med. Assoc..

[42] Ehrchen J.M., Roth J., Barczyk-Kahlert K. (2019). More than suppression: Glucocorticoid action on monocytes and macrophages. Front. Immunol..

[43] Thomas B.J., Porritt R.A., Hertzog P.J., Bardin P.G., Tate M.D. (2014). Glucocorticosteroids enhance replication of respiratory viruses: Effect of adjuvant interferon. Sci. Rep..

[44] Tang X., Feng Y.M., Ni J.X., Zhang J.Y., Liu L.M., Hu K., Wu X.Z., Zhang J.X., Chen J.W., Zhang J.C. (2021). Early Use of Corticosteroid May Prolong SARS-CoV-2 Shedding in Non-Intensive Care Unit Patients with COVID-19 Pneumonia: A Multicenter, Single-Blind, Randomized Control Trial. Respiration.

[45] Russell C.D., Millar J.E., Baillie J.K. (2020). Clinical evidence does not support corticosteroid treatment for 2019-nCoV lung injury. Lancet.

[46] Breakey S., Sharp S.J., Adler A.I., Challis B.G. (2016). Glucocorticoid-induced hyperglycaemia in respiratory disease: A systematic review and meta-analysis. Diabetes Obes. Metab..

[47] Costello R.E., Yimer B.B., Roads P., Jani M., Dixon W.G. (2021). Glucocorticoid use is associated with an increased risk of hypertension. Rheumatology.

[48] Broersen L.H.A., Pereira A.M., Jørgensen J.O.L., Dekkers O.M. (2015). Adrenal Insufficiency in Corticosteroids Use: Systematic Review and Meta-Analysis. J. Clin. Endocrinol. Metab..

[49] Scaroni C., Armigliato M., Cannavò S. (2020). COVID-19 outbreak and steroids administration: Are patients treated for Sars-Cov-2 at risk of adrenal insufficiency?. J. Endocrinol. Investig..

[50] Kaiser U.B., Mirmira R.G., Stewart P.M. (2020). Our Response to COVID-19 as Endocrinologists and Diabetologists. J. Clin. Endocrinol. Metab..

[51] Sheikh A.B., Javed N., Sheikh A.A.E., Upadhyay S., Shekhar R. (2021). Diabetes Insipidus and Concomitant Myocarditis: A Late Sequelae of COVID-19 Infection. J. Investig. Med. High Impact Case Rep..

[52] Rajevac H., Bachan M., Khan Z. (2020). Diabetes insipidus as a symptom of covid-19 infection: Case report. Chest.

[53] L’Heudé M., Poignant S., Elaroussi D., Espitalier F., Ferrandière M., Laffon M. (2019). Nephrogenic diabetes insipidus associated with prolonged sedation with sevoflurane in the intensive care unit. Br. J. Anaesth..

[54] Baldeweg S.E., Ball S., Brooke A., Gleeson H.K., Levy M.J., Prentice M., Wass J. (2018). Society for endocrinology clinical guidance: Inpatient management of cranial diabetes insipidus. Endocr. Connect..

[55] Christ-Crain M., Hoorn E.J., Sherlock M., Thompson C.J., Wass J.A.H. (2020). Endocrinology in the Time of COVID-19: Management of diabetes insipidus and hyponatraemia. Eur. J. Endocrinol..

[56] Dmitrieva N.I., Burg M.B. (2014). Secretion of von Willebrand factor by endothelial cells links sodium to hypercoagulability and thrombosis. Proc. Natl. Acad. Sci. USA.

[57] Miljic D., Miljic P., Doknic M., Pekic S., Stojanovic M., Petakov M., Popovic V. (2014). Adipsic diabetes insipidus and venous thromboembolism (VTE): Recommendations for addressing its hypercoagulability. Hormones.

[58] Iovino M., Messana T., Tortora A., Giusti C., Lisco G., Giagulli V.A., Guastamacchia E., De Pergola G., Triggiani V. (2021). Oxytocin Signaling Pathway: From Cell Biology to Clinical Implications. Endocr. Metab. Immune Disord. Drug Targets.

[59] Soumier A., Sirigu A. (2020). Oxytocin as a potential defence against Covid-19?. Med. Hypotheses.

[60] Diep P.T., Talash K., Kasabri V. (2020). Hypothesis: Oxytocin is a direct COVID-19 antiviral. Med. Hypotheses.

[61] Dekkers O.M., Horváth-Puhó E., Jørgensen J.O.L., Cannegieter S.C., Ehrenstein V., Vandenbroucke J.P., Pereira A.M., Srøensen H.T. (2013). Multisystem morbidity and mortality in cushing’s syndrome: A cohort study. J. Clin. Endocrinol. Metab..

[62] Pivonello R., Isidori A.M., De Martino M.C., Newell-Price J., Biller B.M.K., Colao A. (2016). Complications of Cushing’s syndrome: State of the art. Lancet Diabetes Endocrinol..

[63] Serban A.L., Ferrante E., Carosi G., Indirli R., Arosio M., Mantovani G. (2021). COVID-19 in Cushing disease: Experience of a single tertiary centre in Lombardy. J. Endocrinol. Investig..

[64] Belaya Z., Golounina O., Melnichenko G., Tarbaeva N., Pashkova E., Gorokhov M., Kalashnikov V., Dzeranova L., Fadeev V., Volchkov P. (2021). Clinical course and outcome of patients with ACTH-dependent Cushing’s syndrome infected with novel coronavirus disease-19 (COVID-19): Case presentations. Endocrine.

[65] Beretta F., Dassie F., Parolin M., Boscari F., Barbot M., Busetto L., Mioni R., De Carlo E., Scaroni C., Fallo F. (2020). Practical Considerations for the Management of Cushing’s Disease and COVID-19: A Case Report. Front. Endocrinol..

[66] Fleseriu M., Biller B.M.K., Findling J.W., Molitch M.E., Schteingart D.E., Gross C. (2012). Mifepristone, a glucocorticoid receptor antagonist, produces clinical and metabolic benefits in patients with Cushing’s syndrome. J. Clin. Endocrinol. Metab..

[67] Aitken A.E., Morgan E.T. (2007). Gene-specific effects of inflammatory cytokines on cytochrome P450 2C, 2B6 and 3A4 mRNA levels in human hepatocytes. Drug Metab. Dispos..

[68] McCreary E.K., Pogue J.M. (2020). Coronavirus disease 2019 treatment: A review of early and emerging options. Open Forum Infect. Dis..

[69] Yuen K.C.J. (2021). Growth hormone deficiency, acromegaly and COVID-19: Transitioning from media reports to knowledge and a growth hormone hypothesis. Growth Horm. IGF Res..

[70] Isgaard J., Arcopinto M., Karason K., Cittadini A. (2014). GH and the cardiovascular system: An update on a topic at heart. Endocrine.

[71] Chanson P. (2020). The heart in growth hormone (GH) deficiency and the cardiovascular effects of GH. Ann. Endocrinol..

[72] Lombardi G., Di Somma C., Grasso L.F.S., Savanelli M.C., Colao A., Pivonello R. (2012). The cardiovascular system in growth hormone excess and growth hormone deficiency. J. Endocrinol. Investig..

[73] Giustina A., Legg E., Cesana B.M., Frara S., Mortini P., Fleseriu M. (2021). Results from ACROCOVID: An international survey on the care of acromegaly during the COVID-19 era. Endocrine.

[74] Fleseriu M., Dekkers O.M., Karavitaki N. (2020). ENDOCRINOLOGY in the TIME of COVID-19: Management of pituitary tumours. Eur. J. Endocrinol..

[75] Fleseriu M., Buchfelder M., Cetas J.S., Fazeli P.K., Mallea-Gil S.M., Gurnell M., McCormack A., Pineyro M.M., Syro L.V., Tritos N.A. (2020). Pituitary society guidance: Pituitary disease management and patient care recommendations during the COVID-19 pandemic—An international perspective. Pituitary.

[76] Mitchell R.A., King J.A.J., Goldschlager T., Wang Y.Y. (2020). Impact of COVID-19 on pituitary surgery. ANZ J. Surg..

[77] Penner F., Grottoli S., Lanotte M.M.R., Garbossa D., Zenga F. (2020). Pituitary surgery during Covid-19: A first hand experience and evaluation. J. Endocrinol. Investig..

[78] Arlt W., Baldeweg S.E., Pearce S.H.S., Simpson H.L. (2020). ENDOCRINOLOGY in the TIME of COVID-19: Management of adrenal insufficiency. Eur. J. Endocrinol..

[79] Hariyanto T.I., Kurniawan A. (2020). Thyroid disease is associated with severe coronavirus disease 2019 (COVID-19) infection. Diabetes Metab. Syndr. Clin. Res. Rev..

[80] Scappaticcio L., Pitoia F., Esposito K., Piccardo A., Trimboli P. (2020). Impact of COVID-19 on the thyroid gland: An update. Rev. Endocr. Metab. Disord..

[81] Lisco G., De Tullio A., Jirillo E., Giagulli V.A., De Pergola G., Guastamacchia E., Triggiani V. (2021). Thyroid and COVID-19: A review on pathophysiological, clinical and organizational aspects. J. Endocrinol. Investig..

[82] Campos-Barrera E., Alvarez-Cisneros T., Davalos-Fuentes M. (2020). Subacute Thyroiditis Associated with COVID-19. Case Rep. Endocrinol..

[83] Brancatella A., Ricci D., Cappellani D., Viola N., Sgrò D., Santini F., Latrofa F. (2020). Is subacute thyroiditis an underestimated manifestation of SARS-CoV-2 infection? insights from a case series. J. Clin. Endocrinol. Metab..

[84] Ruggeri R.M., Campennì A., Siracusa M., Frazzetto G., Gullo D. (2020). Subacute thyroiditis in a patient infected with SARS-COV-2: An endocrine complication linked to the COVID-19 pandemic. Hormones.

[85] Asfuroglu Kalkan E., Ates I. (2020). A case of subacute thyroiditis associated with Covid-19 infection. J. Endocrinol. Investig..

[86] Gorini F., Bianchi F., Iervasi G. (2020). Covid-19 and thyroid: Progress and prospects. Int. J. Environ. Res. Public Health.

[87] Rotondi M., Coperchini F., Ricci G., Denegri M., Croce L., Ngnitejeu S.T., Villani L., Magri F., Latrofa F., Chiovato L. (2020). Detection of SARS-COV-2 receptor ACE-2 mRNA in thyroid cells: A clue for COVID-19-related subacute thyroiditis. J. Endocrinol. Investig..

[88] Ippolito S., Dentali F., Tanda M.L. (2020). SARS-CoV-2: A potential trigger for subacute thyroiditis? Insights from a case report. J. Endocrinol. Investig..

[89] Mattar S.A.M., Koh S.J.Q., Rama Chandran S., Cherng B.P.Z. (2020). Subacute thyroiditis associated with COVID-19. BMJ Case Rep..

[90] Bellastella G., Maiorino M.I., Esposito K. (2020). Endocrine complications of COVID-19: What happens to the thyroid and adrenal glands?. J. Endocrinol. Investig..

[91] Tee L.Y., Hajanto S., Rosario B.H. (2020). COVID-19 complicated by Hashimoto’s thyroiditis. Singap. Med. J..

[92] Murtas R., Andreano A., Gervasi F., Guido D., Consolazio D., Tunesi S., Andreoni L., Greco M.T., Gattoni M.E., Sandrini M. (2020). Association between autoimmune diseases and COVID-19 as assessed in both a test-negative case-control and population case-control design. Autoimmun. Highlights.

[93] Mateu-Salat M., Urgell E., Chico A. (2020). SARS-COV-2 as a trigger for autoimmune disease: Report of two cases of Graves’ disease after COVID-19. J. Endocrinol. Investig..

[94] Lania A., Sandri M.T., Cellini M., Mirani M., Lavezzi E., Mazziotti G. (2020). Thyrotoxicosis in patients with COVID-19: The THYRCOV study. Eur. J. Endocrinol..

[95] Caron P. (2020). Thyroid disorders and SARS-CoV-2 infection: From pathophysiological mechanism to patient management. Ann. Endocrinol..

[96] Davies T.F. (2008). Infection and Autoimmune Thyroid Disease. J. Clin. Endocrinol. Metab..

[97] Antunes T.T., Gagnon A., Chen B., Pacini F., Smith T.J., Sorisky A. (2006). Interleukin-6 release from human abdominal adipose cells is regulated by thyroid-stimulating hormone: Effect of adipocyte differentiation and anatomic depot. Am. J. Physiol. Endocrinol. Metab..

[98] Antunes T.T., Gagnon A.M., Langille M.L., Sorisky A. (2008). Thyroid-stimulating hormone induces interleukin-6 release from human adipocytes through activation of the nuclear factor-κB pathway. Endocrinology.

[99] Bell A., Gagnon A.M., Sorisky A. (2003). TSH stimulates IL-6 secretion from adipocytes in culture. Arterioscler. Thromb. Vasc. Biol..

[100] Contreras-Jurado C., Alonso-Merino E., Saiz-Ladera C., Valinõ A.J., Regadera J., Alemany S., Aranda A. (2016). The Thyroid Hormone Receptors Inhibit Hepatic Interleukin-6 Signaling during Endotoxemia. Sci. Rep..

[101] van Gerwen M., Alsen M., Little C., Barlow J., Naymagon L., Tremblay D., Sinclair C.F., Genden E. (2020). Outcomes of Patients With Hypothyroidism and COVID-19: A Retrospective Cohort Study. Front. Endocrinol..

[102] Brix T.H., Hegedüs L., Hallas J., Lund L.C. (2021). Risk and course of SARS-CoV-2 infection in patients treated for hypothyroidism and hyperthyroidism. Lancet Diabetes Endocrinol..

[103] Chen M., Zhou W., Xu W. (2020). Thyroid Function Analysis in 50 Patients with COVID-19: A Retrospective Study. Thyroid.

[104] Davies P.H., Black E.G., Sheppard M.C., Franklyn J.A. (1996). Relation between serum interleukin-6 and thyroid hormone concentrations in 270 hospital in-patients with non-thyroidal illness. Clin. Endocrinol..

[105] Foks M., Dudek A., Polok K., Nowak-Kózka I., Fronczek J., Szczeklik W. (2019). Thyroid hormones as potential prognostic factors in sepsis. Anaesthesiol. Intensive Ther..

[106] Chang X., Zhang S., Zhang M., Wang H., Fan C., Gu Y., Wei J., Qiu C. (2018). Free triiodothyronine and global registry of acute coronary events risk score on predicting long-term major adverse cardiac events in STEMI patients undergoing primary PCI. Lipids Health Dis..

[107] Triggiani V., Iacoviello M. (2013). Thyroid Disorders in Chronic Heart Failure: From Prognostic Set-up to Therapeutic Management. Endocrine, Metab. Immune Disord. Targets.

[108] Lisco G., De Tullio A., Iacoviello M., Triggiani V. (2020). Congestive Heart Failure and Thyroid Dysfunction: The Role of the Low T3 Syndrome and Therapeutic Aspects. Endocr. Metab. Immune Disord. Drug Targets.

[109] Rizzo C., Gioia M.I., Parisi G., Triggiani V., Iacoviello M. (2018). Dysthyroidism and chronic heart failure: Pathophysiological mechanisms and therapeutic approaches. Advances in Experimental Medicine and Biology.

[110] Pingitore A., Galli E., Barison A., Iervasi A., Scarlattini M., Nucci D., L’Abbate A., Mariotti R., Iervasi G. (2008). Acute Effects of Triiodothyronine (T_3_) Replacement Therapy in Patients with Chronic Heart Failure and Low-T _3_ Syndrome: A Randomized, Placebo-Controlled Study. J. Clin. Endocrinol. Metab..

[111] Gao W., Guo W., Guo Y., Shi M., Dong G., Wang G., Ge Q., Zhu J., Zhou X. (2020). Thyroid hormone concentrations in severely or critically ill patients with COVID-19. J. Endocrinol. Investig..

[112] Lui D.T.W., Lee C.H., Chow W.S., Lee A.C.H., Tam A.R., Fong C.H.Y., Law C.Y., Leung E.K.H., To K.K.W., Tan K.C.B. (2021). Role of Non-Thyroidal Illness Syndrome in Predicting Adverse Outcomes in COVID-19 Patients Predominantly of Mild to Moderate Severity. Clin. Endocrinol..

[113] Almaghlouth N.K., Davis M.G., Davis M.A., Anyiam F.E., Guevara R., Antony S.J. (2020). Risk factors for mortality among patients with SARS-CoV-2 infection: A longitudinal observational study. J. Med. Virol..

[114] Ulhaq Z.S., Soraya G.V. (2020). Interleukin-6 as a potential biomarker of COVID-19 progression. Med. Mal. Infect..

[115] Henry B.M., de Oliveira M.H.S., Benoit S., Plebani M., Lippi G. (2020). Hematologic, biochemical and immune biomarker abnormalities associated with severe illness and mortality in coronavirus disease 2019 (COVID-19): A meta-analysis. Clin. Chem. Lab. Med..

[116] Pantos C., Kostopanagiotou G., Armaganidis A., Trikas A., Tseti I., Mourouzis I. (2020). Triiodothyronine for the treatment of critically ill patients with COVID-19 infection: A structured summary of a study protocol for a randomised controlled trial. Trials.

[117] Jiménez-Blanco S., Pla-Peris B., Marazuela M. (2020). COVID-19: A cause of recurrent Graves’ hyperthyroidism?. J. Endocrinol. Investig..

[118] Jyonouchi S.C., Valyasevi R.W., Harteneck D.A., Dutton C.M., Bahn R.S. (2001). Interleukin-6 stimulates thyrotropin receptor expression in human orbital preadipocyte fibroblasts from patients with Graves’ ophthalmopathy. Thyroid.

[119] Krieger C.C., Perry J.D., Morgan S.J., Kahaly G.J., Gershengorn M.C. (2017). TSH/IGF-1 receptor cross-Talk rapidly activates extracellular signal-regulated kinases in multiple cell types. Endocrinology.

[120] Winn B.J. (2020). Is there a role for insulin-like growth factor inhibition in the treatment of COVID-19-related adult respiratory distress syndrome?. Med. Hypotheses.

[121] Squizzato A., Romualdi E., Büller H.R., Gerdes V.E.A. (2007). Clinical review: Thyroid dysfunction and effects on coagulation and fibrinolysis: A systematic review. J. Clin. Endocrinol. Metab..

[122] Kahaly G.J., Dillmann W.H. (2005). Thyroid hormone action in the heart. Endocr. Rev..

[123] Rubingh J., van der Spek A., Fliers E., Boelen A. (2020). The role of thyroid hormone in the innate and adaptive immune response during infection. Compr. Physiol..

[124] Boelaert K., Edward Visser W., Taylor P.N., Moran C., Léger J., Persani L. (2020). ENDOCRINOLOGY in the TIME of COVID-19: Management of hyperthyroidism and hypothyroidism. Eur. J. Endocrinol..

[125] Kahaly G.J. (2020). Management of Graves Thyroidal and Extrathyroidal Disease: An Update. J. Clin. Endocrinol. Metab..

[126] Kobaly K., Mandel S.J., Cappola A.R., Kim C.S. (2020). Letter to the Editor: “Our Response to COVID-19 as Endocrinologists and Diabetologists. ” J. Clin. Endocrinol. Metab..

[127] Vicente N., Cardoso L., Barros L., Carrilho F. (2017). Antithyroid Drug-Induced Agranulocytosis: State of the Art on Diagnosis and Management. Drugs R D.

[128] Vigliar E., Iaccarino A., Bruzzese D., Malapelle U., Bellevicine C., Troncone G. (2021). Cytology in the time of coronavirus disease (covid-19): An Italian perspective. J. Clin. Pathol..

[129] Venessa H.M.T., Matti G., Anthony G., Roderick C.B., Bruce G.R. (2020). Thyroid cancer in the age of COVID-19. Endocr. Relat. Cancer.

[130] Shaha A.R. (2020). Thyroid surgery during COVID-19 pandemic: Principles and philosophies. Head and Neck.

[131] Lombardi C.P., D’Amore A., Grani G., Ramundo V., Boscherini M., Gordini L., Marzi F., Tedesco S., Bocale R. (2020). Endocrine surgery during COVID-19 pandemic: Do we need an update of indications in Italy?. Endocrine.

[132] Jozaghi Y., Zafereo M.E., Perrier N.D., Wang J.R., Grubbs E., Gross N.D., Fisher S., Sturgis E.M., Goepfert R.P., Lai S.Y. (2020). Endocrine surgery in the Coronavirus disease 2019 pandemic: Surgical Triage Guidelines. Head and Neck.

[133] Baud G., Brunaud L., Lifante J.C., Tresallet C., Sebag F., Bizard J.P., Mathonnet M., Menegaux F., Caiazzo R., Mirallié E. (2020). Endocrine surgery during and after the COVID-19 epidemic: Expert guidelines from AFCE. J. Visc. Surg..

[134] Pacini F., Basolo F., Bellantone R., Boni G., Cannizzaro M.A., De Palma M., Durante C., Elisei R., Fadda G., Frasoldati A. (2018). Italian consensus on diagnosis and treatment of differentiated thyroid cancer: Joint statements of six Italian societies. J. Endocrinol. Investig..

[135] Kahaly G.J., Bartalena L., Hegedüs L., Leenhardt L., Poppe K., Pearce S.H. (2018). 2018 European thyroid association guideline for the management of graves’ hyperthyroidism. Eur. Thyroid J..

[136] Yano Y., Sugino K., Akaishi J., Uruno T., Okuwa K., Shibuya H., Kitagawa W., Nagahama M., Ito K., Ito K. (2011). Treatment of autonomously functioning thyroid nodules at a single institution: Radioiodine therapy, surgery, and ethanol injection therapy. Ann. Nucl. Med..

[137] Freudenberg L.S., Paez D., Giammarile F., Cerci J., Modiselle M., Pascual T.N.B., El-Haj N., Orellana P., Pynda Y., Carrió I. (2020). Global impact of COVID-19 on nuclear medicine departments: An international survey in April 2020. J. Nucl. Med..

[138] Millet J.K., Whittaker G.R. (2018). Physiological and molecular triggers for SARS-CoV membrane fusion and entry into host cells. Virology.

[139] Charoenngam N., Holick M.F. (2020). Immunologic effects of vitamin d on human health and disease. Nutrients.

[140] Bouillon R., Marcocci C., Carmeliet G., Bikle D., White J.H., Dawson-Hughes B., Lips P., Munns C.F., Lazaretti-Castro M., Giustina A. (2019). Skeletal and Extraskeletal Actions of Vitamin D: Current Evidence and Outstanding Questions. Endocr. Rev..

[141] Wacker M., Holiack M.F. (2013). Vitamin D-effects on skeletal and extraskeletal health and the need for supplementation. Nutrients.

[142] Nair R., Maseeh A. (2012). Vitamin D: The sunshine vitamin. J. Pharmacol. Pharmacother..

[143] Holick M.F. (2007). Medical progress: Vitamin D deficiency. N. Engl. J. Med..

[144] Lips P., Cashman K.D., Lamberg-Allardt C., Bischoff-Ferrari H.A., Obermayer-Pietsch B., Bianchi M.L., Stepan J., Fuleihan G.E.H., Bouillon R. (2019). Current Vitamin D status in European and Middle East countries and strategies to prevent Vitamin D deficiency: A position statement of the European Calcified Tissue Society. Eur. J. Endocrinol..

[145] Kim D. (2017). The role of vitamin D in thyroid diseases. Int. J. Mol. Sci..

[146] Miteva M.Z., Nonchev B.I., Orbetzova M.M., Stoencheva S.D. (2020). Vitamin D and Autoimmune Thyroid Diseases–A Review. Folia Med..

[147] Aranow C. (2011). Vitamin D and the immune system. J. Investig. Med..

[148] Boonstra A., Barrat F.J., Crain C., Heath V.L., Savelkoul H.F.J., O’Garra A. (2001). 1α,25-Dihydroxyvitamin D3 Has a Direct Effect on Naive CD4 + T Cells to Enhance the Development of Th2 Cells. J. Immunol..

[149] Fisher S.A., Rahimzadeh M., Brierley C., Gration B., Doree C., Kimber C.E., Cajide A.P., Lamikanra A.A., Roberts D.J. (2019). The role of Vitamin D in increasing circulating T regulatory cell numbers and modulating T regulatory cell phenotypes in patients with inflammatory disease or in healthy volunteers: A systematic review. PLoS ONE.

[150] Hewison M. (2010). Vitamin D and the immune system: New perspectives on an old theme. Endocrinol. Metab. Clin. N. Am..

[151] Medrano M., Carrillo-Cruz E., Montero I., Perez-Simon J.A. (2018). Vitamin D: Effect on haematopoiesis and immune system and clinical applications. Int. J. Mol. Sci..

[152] Lang C.-L., Wang M.-H., Chiang C.-K., Lu K.-C. (2014). Vitamin D and the Immune System from the Nephrologist’s Viewpoint. ISRN Endocrinol..

[153] Chang S.H., Chung Y., Dong C. (2010). Vitamin D suppresses Th17 cytokine production by inducing C/EBP Homologous Protein (CHOP) expression. J. Biol. Chem..

[154] Hayes C.E., Hubler S.L., Moore J.R., Barta L.E., Praska C.E., Nashold F.E. (2015). Vitamin D actions on CD4+ T cells in autoimmune disease. Front. Immunol..

[155] Iyer S.S., Cheng G. (2012). Role of interleukin 10 transcriptional regulation in inflammation and autoimmune disease. Crit. Rev. Immunol..

[156] Tramontana F., Napoli N., El-Hajj Fuleihan G., Strollo R. (2020). The D-side of COVID-19: Musculoskeletal benefits of vitamin D and beyond. Endocrine.

[157] Martineau A.R., Jolliffe D.A., Hooper R.L., Greenberg L., Aloia J.F., Bergman P., Dubnov-Raz G., Esposito S., Ganmaa D., Ginde A.A. (2017). Vitamin D supplementation to prevent acute respiratory tract infections: Systematic review and meta-analysis of individual participant data. BMJ.

[158] Silberstein M. (2020). Vitamin D: A simpler alternative to tocilizumab for trial in COVID-19?. Med. Hypotheses.

[159] Grant W.B., Lahore H., McDonnell S.L., Baggerly C.A., French C.B., Aliano J.L., Bhattoa H.P. (2020). Evidence that vitamin d supplementation could reduce risk of influenza and covid-19 infections and deaths. Nutrients.

[160] Mitchell F. (2020). Vitamin-D and COVID-19: Do deficient risk a poorer outcome?. Lancet Diabetes Endocrinol..

[161] Booth C.M., Matukas L.M., Tomlinson G.A., Rachlis A.R., Rose D.B., Dwosh H.A., Walmsley S.L., Mazzulli T., Avendano M., Derkach P. (2003). Clinical Features and Short-term Outcomes of 144 Patients with SARS in the Greater Toronto Area. J. Am. Med. Assoc..

[162] Sun J.K., Zhang W.H., Zou L., Liu Y., Li J.J., Kan X.H., Dai L., Shi Q.K., Yuan S.T., Yu W.K. (2020). Serum calcium as a biomarker of clinical severity and prognosis in patients with coronavirus disease 2019. Aging.

[163] Di Filippo L., Formenti A.M., Rovere-Querini P., Carlucci M., Conte C., Ciceri F., Zangrillo A., Giustina A. (2020). Hypocalcemia is highly prevalent and predicts hospitalization in patients with COVID-19. Endocrine.

[164] Steele T., Kolamunnage-Dona R., Downey C., Toh C.H., Welters I. (2013). Assessment and clinical course of hypocalcemia in critical illness. Crit. Care.

[165] Martha J.W., Wibowo A., Pranata R. (2021). Hypocalcemia is associated with severe COVID-19: A systematic review and meta-analysis. Diabetes Metab. Syndr. Clin. Res. Rev..

[166] Bennouar S., Cherif A.B., Kessira A., Bennouar D.E., Abdi S. (2020). Vitamin D Deficiency and Low Serum Calcium as Predictors of Poor Prognosis in Patients with Severe COVID-19. J. Am. Coll. Nutr..

[167] Brandão C.M.Á., Chiamolera M.I., Biscolla R.P.M., Lima J.V., De Francischi Ferrer C.M., Prieto W.H., de Sá Tavares Russo P., de Sá J., Dos Santos Lazari C., Granato C.F.H. (2021). No association between vitamin D status and COVID-19 infection in São Paulo, Brazil. Arch. Endocrinol. Metab..

[168] Marik P.E., Kory P., Varon J. (2020). Does vitamin D status impact mortality from SARS-CoV-2 infection?. Med. Drug Discov..

[169] Pal R., Ram S., Zohmangaihi D., Biswas I., Suri V., Yaddanapudi L.N., Malhotra P., Soni S.L., Puri G.D., Bhalla A. (2021). High Prevalence of Hypocalcemia in Non-severe COVID-19 Patients: A Retrospective Case-Control Study. Front. Med..

[170] Carpagnano G.E., Di Lecce V., Quaranta V.N., Zito A., Buonamico E., Capozza E., Palumbo A., Di Gioia G., Valerio V.N., Resta O. (2020). Vitamin D deficiency as a predictor of poor prognosis in patients with acute respiratory failure due to COVID-19. J. Endocrinol. Investig..

[171] L Bishop E., Ismailova A., Dimeloe S., Hewison M., White J.H. (2021). Vitamin D and Immune Regulation: Antibacterial, Antiviral, Anti-Inflammatory. JBMR Plus.

[172] Munshi R., Hussein M.H., Toraih E.A., Elshazli R.M., Jardak C., Sultana N., Youssef M.R., Omar M., Attia A.S., Fawzy M.S. (2021). Vitamin D insufficiency as a potential culprit in critical COVID-19 patients. J. Med. Virol..

[173] Efficacy of Vitamin D Supplementation to Prevent the Risk of Acquiring COVID-19 in Healthcare Workers–Full Text View–ClinicalTrials.gov. https://clinicaltrials.gov/ct2/show/NCT04535791.

[174] Prevention and Treatment with Calcifediol of COVID-19 Induced Acute Respiratory Syndrome–Full Text View–ClinicalTrials.gov. https://clinicaltrials.gov/ct2/show/NCT04366908.

[175] Cesareo R., Attanasio R., Caputo M., Castello R., Chiodini I., Falchetti A., Guglielmi R., Papini E., Santonati A., Scillitani A. (2018). Italian association of clinical endocrinologists (AME) and Italian chapter of the American association of clinical endocrinologists (AACE) position statement: Clinical management of vitamin D deficiency in adults. Nutrients.

[176] Bialek S., Boundy E., Bowen V., Chow N., Cohn A., Dowling N., Ellington S., Gierke R., Hall A., MacNeil J. (2020). Severe Outcomes Among Patients with Coronavirus Disease 2019 (COVID-19)—United States, February 12–16 March 2020. MMWR. Morb. Mortal. Wkly. Rep..

[177] Palaiodimos L., Kokkinidis D.G., Li W., Karamanis D., Ognibene J., Arora S., Southern W.N., Mantzoros C.S. (2020). Severe obesity is associated with higher in-hospital mortality in a cohort of patients with COVID-19 in the Bronx, New York. Metabolism..

[178] Simonnet A., Chetboun M., Poissy J., Raverdy V., Noulette J., Duhamel A., Labreuche J., Mathieu D., Pattou F., Jourdain M. (2020). High prevalence of obesity in severe acute respiratory syndrome coronavirus-2 (SARS-CoV-2) requiring invasive mechanical ventilation. Obesity.

[179] Giagulli V.A., Guastamacchia E., Magrone T., Jirillo E., Lisco G., De Pergola G., Triggiani V. (2020). Worse progression of COVID-19 in men: Is Testosterone a key factor?. Andrology.

[180] Lisco G., De Tullio A., Giagulli V.A., Guastamacchia E., De Pergola G., Triggiani V. (2020). Hypothesized mechanisms explaining poor prognosis in type 2 diabetes patients with COVID-19: A review. Endocrine.

[181] Bassatne A., Chakhtoura M., Saad R., Fuleihan G.E.H. (2019). Vitamin D supplementation in obesity and during weight loss: A review of randomized controlled trials. Metabolism.

[182] Turner J., Gittoes N., Selby P. (2016). Emergency management of acute hypocalcaemia in adult patients. Endocr. Connect..

[183] Gittoes N.J., Criseno S., Appelman-Dijkstra N.M., Bollerslev J., Canalis E., Rejnmark L., Hassan-Smith Z. (2020). Management of calcium metabolic disorders and osteoporosis. Eur. J. Endocrinol..

[184] Renaghan A.D., Rosner M.H. (2018). Hypercalcemia: Etiology and management. Nephrol. Dial. Transplant..

[185] Walsh J., Gittoes N., Selby P. (2016). Emergency management of acute hypercalcaemia in adult patients. Endocr. Connect..

[186] Yu E.W., Tsourdi E., Clarke B.L., Bauer D.C., Drake M.T. (2020). Osteoporosis Management in the Era of COVID -19. J. Bone Miner. Res..

[187] Tu K.N., Lie J.D., Wan C.K.V., Cameron M., Austel A.G., Nguyen J.K., Van K., Hyun D. (2018). Osteoporosis: A review of treatment options. Pharm. Ther..

[188] Ott S.M. (2005). Long-Term Safety of Bisphosphonates. J. Clin. Endocrinol. Metab..

[189] Lyu H., Yoshida K., Zhao S.S., Wei J., Zeng C., Tedeschi S.K., Leder B.Z., Lei G., Tang P., Solomon D.H. (2020). Delayed Denosumab Injections and Fracture Risk Among Patients With Osteoporosis: A Population-Based Cohort Study. Ann. Intern. Med..

[190] Tsourdi E., Langdahl B., Cohen-Solal M., Aubry-Rozier B., Eriksen E.F., Guañabens N., Obermayer-Pietsch B., Ralston S.H., Eastell R., Zillikens M.C. (2017). Discontinuation of Denosumab therapy for osteoporosis: A systematic review and position statement by ECTS. Bone.

[191] Ono T., Hayashi M., Sasaki F., Nakashima T. (2020). RANKL biology: Bone metabolism, the immune system, and beyond. Inflamm. Regen..

[192] Bone H.G., Bolognese M.A., Yuen C.K., Kendler D.L., Wang H., Liu Y., San Martin J. (2008). Effects of Denosumab on Bone Mineral Density and Bone Turnover in Postmenopausal Women. J. Clin. Endocrinol. Metab..

[193] Eastell R., Rosen C.J., Black D.M., Cheung A.M., Murad M.H., Shoback D. (2019). Pharmacological management of osteoporosis in postmenopausal women: An endocrine society clinical practice guideline. J. Clin. Endocrinol. Metab..

[194] Black D.M., Delmas P.D., Eastell R., Reid I.R., Boonen S., Cauley J.A., Cosman F., Lakatos P., Leung P.C., Man Z. (2007). Once-Yearly Zoledronic Acid for Treatment of Postmenopausal Osteoporosis. N. Engl. J. Med..

[195] Lindsay R., Krege J.H., Marin F., Jin L., Stepan J.J. (2016). Teriparatide for osteoporosis: Importance of the full course. Osteoporos. Int..

[196] Minisola S., Cipriani C., Grotta G.D., Colangelo L., Occhiuto M., Biondi P., Sonato C., Vigna E., Cilli M., Pepe J. (2019). Update on the safety and efficacy of teriparatide in the treatment of osteoporosis. Ther. Adv. Musculoskelet. Dis..

[197] Kumar Jain V., Lal H., Kumar Patralekh M., Vaishya R. (2020). Fracture management during COVID-19 pandemic: A systematic review. J. Clin. Orthop. Trauma.

[198] Napoli N., Elderkin A.L., Kiel D.P., Khosla S. (2020). Managing fragility fractures during the COVID-19 pandemic. Nat. Rev. Endocrinol..

[199] AACE Position Statement: Coronavirus (COVID-19) and People with Adrenal Insufficiency and Cushing’s Syndrome|American Association of Clinical Endocrinology. https://pro.aace.com/recent-news-and-updates/aace-position-statement-coronavirus-covid-19-and-people-adrenal.

[200] COVID-19 Adrenal Crisis Information|Society for Endocrinology. https://www.endocrinology.org/clinical-practice/clinical-guidance/adrenal-crisis/covid-19-adrenal-crisis-information/.

[201] Isidori A.M., Arnaldi G., Boscaro M., Falorni A., Giordano C., Giordano R., Pivonello R., Pofi R., Hasenmajer V., Venneri M.A. (2020). COVID-19 infection and glucocorticoids: Update from the Italian Society of Endocrinology Expert Opinion on steroid replacement in adrenal insufficiency. J. Endocrinol. Investig..

[202] Tresoldi A.S., Sumilo D., Perrins M., Toulis K.A., Prete A., Reddy N., Wass J.A.H., Arlt W., Nirantharakumar K. (2020). Increased Infection Risk in Addison’s Disease and Congenital Adrenal Hyperplasia. J. Clin. Endocrinol. Metab..

[203] Bergthorsdottir R., Leonsson-Zachrisson M., Odén A., Johannsson G. (2006). Premature mortality in patients with Addison’s disease: A population-based study. J. Clin. Endocrinol. Metab..

[204] Erichsen M.M., Løvås K., Fougner K.J., Svartberg J., Hauge E.R., Bollerslev J., Berg J.P., Mella B., Husebye E.S. (2009). Normal overall mortality rate in Addison’s disease, but young patients are at risk of premature death. Eur. J. Endocrinol..

[205] Bancos I., Hazeldine J., Chortis V., Hampson P., Taylor A.E., Lord J.M., Arlt W. (2017). Primary adrenal insufficiency is associated with impaired natural killer cell function: A potential link to increased mortality. Eur. J. Endocrinol..

[206] Edvardsen K., Bjånesøy T., Hellesen A., Breivik L., Bakke M., Husebye E.S., Bratland E. (2015). Peripheral Blood Cells from Patients with Autoimmune Addison’s Disease Poorly Respond to Interferons in Vitro, Despite Elevated Serum Levels of Interferon-Inducible Chemokines. J. Interf. Cytokine Res..

[207] Harris P., Bennett A., Schoeller T., Otto-Schoeller A., Wechselberger G. (2001). Waterhouse–Friderichsen Syndrome. N. Engl. J. Med..

[208] Hellesen A., Bratland E. (2019). The potential role for infections in the pathogenesis of autoimmune Addison’s disease. Clin. Exp. Immunol..

[209] Piticchio T., Le Moli R., Tumino D., Frasca F. (2021). Relationship between betacoronaviruses and the endocrine system: A new key to understand the COVID-19 pandemic—A comprehensive review. J. Endocrinol. Investig..

[210] Hashim M., Athar S., Gaba W.H. (2021). New onset adrenal insufficiency in a patient with COVID-19. BMJ Case Rep..

[211] Heidarpour M., Vakhshoori M., Abbasi S., Shafie D., Rezaei N. (2020). Adrenal insufficiency in coronavirus disease 2019: A case report. J. Med. Case Rep..

[212] Iuga A.C., Marboe C.C., Yilmaz M.M., Lefkowitch J.H., Gauran C., Lagana S.M. (2020). Adrenal vascular changes in COVID-19 autopsies. Arch. Pathol. Lab. Med..

[213] Porfidia A., Pola R. (2020). Venous thromboembolism in COVID-19 patients. J. Thromb. Haemost..

[214] Frankel M., Feldman I., Levine M., Frank Y., Bogot N.R., Benjaminov O., Kurd R., Breuer G.S., Munter G. (2020). Bilateral adrenal hemorrhage in coronavirus disease 2019 patient: A case report. J. Clin. Endocrinol. Metab..

[215] Terpos E., Ntanasis-Stathopoulos I., Elalamy I., Kastritis E., Sergentanis T.N., Politou M., Psaltopoulou T., Gerotziafas G., Dimopoulos M.A. (2020). Hematological findings and complications of COVID-19. Am. J. Hematol..

[216] Tavakolpour S., Rakhshandehroo T., Wei E.X., Rashidian M. (2020). Lymphopenia during the COVID-19 infection: What it shows and what can be learned. Immunol. Lett..

[217] Panesar N.S., Lam C.W.K., Chan M.H.M., Wong C.K., Sung J.J.Y. (2004). Lymphopenia and neutrophilia in SARS are related to the prevailing serum cortisol (2). Eur. J. Clin. Investig..

[218] Hahner S., Loeffler M., Bleicken B., Drechsler C., Milovanovic D., Fassnacht M., Ventz M., Quinkler M., Allolio B. (2010). Epidemiology of adrenal crisis in chronic adrenal insufficiency: The need for new prevention strategies. Eur. J. Endocrinol..

[219] Hahner S., Spinnler C., Fassnacht M., Burger-Stritt S., Lang K., Milovanovic D., Beuschlein F., Willenberg H.S., Quinkler M., Allolio B. (2015). High incidence of adrenal crisis in educated patients with chronic adrenal insufficiency: A prospective study. J. Clin. Endocrinol. Metab..

[220] De Leo M., Pivonello R., Auriemma R.S., Cozzolino A., Vitale P., Simeoli C., De Martino M.C., Lombardi G., Colao A. (2010). Cardiovascular disease in Cushing’s syndrome: Heart versus vasculature. Neuroendocrinol. Neuroendocrinol..

[221] Chanson P., Salenave S. (2010). Metabolic syndrome in Cushing’s syndrome. Neuroendocrinol. Neuroendocrinol..

[222] Trementino L., Arnaldi G., Appolloni G., Daidone V., Scaroni C., Casonato A., Boscaro M. (2010). Coagulopathy in Cushing’s syndrome. Neuroendocrinol. Neuroendocrinol..

[223] Hasenmajer V., Sbardella E., Sciarra F., Minnetti M., Isidori A.M., Venneri M.A. (2020). The Immune System in Cushing’s Syndrome. Trends Endocrinol. Metab..

[224] Broersen L.H.A., Jha M., Biermasz N.R., Pereira A.M., Dekkers O.M. (2018). Effectiveness of medical treatment for Cushing’s syndrome: A systematic review and meta-analysis. Pituitary.

[225] Braun L.T., Rubinstein G., Zopp S., Vogel F., Schmid-Tannwald C., Escudero M.P., Honegger J., Ladurner R., Reincke M. (2020). Recurrence after pituitary surgery in adult Cushing’s disease: A systematic review on diagnosis and treatment. Endocrine.

[226] Newell-Price J., Nieman L.K., Reincke M., Tabarin A. (2020). ENDOCRINOLOGY in the TIME of COVID-19: Management of Cushing’s syndrome. Eur. J. Endocrinol..

[227] Nieman L.K., Biller B.M.K., Findling J.W., Newell-Price J., Savage M.O., Stewart P.M., Montori V.M., Edwards H. (2008). The diagnosis of Cushing’s syndrome: An endocrine society clinical practice guideline. J. Clin. Endocrinol. Metab..

[228] Fassnacht M., Arlt W., Bancos I., Dralle H., Newell-Price J., Sahdev A., Tabarin A., Terzolo M., Tsagarakis S., Dekkers O.M. (2016). Management of adrenal incidentalomas: European Society of Endocrinology Clinical Practice Guideline in collaboration with the European Network for the Study of Adrenal Tumors. Eur. J. Endocrinol..

[229] Young J., Bertherat J., Vantyghem M.C., Chabre O., Senoussi S., Chadarevian R., Castinetti F. (2018). Hepatic safety of ketoconazole in Cushing’s syndrome: Results of a Compassionate Use Programme in France. Eur. J. Endocrinol..

[230] Pivonello R., Ferrigno R., Isidori A.M., Biller B.M.K., Grossman A.B., Colao A. (2020). COVID-19 and Cushing’s syndrome: Recommendations for a special population with endogenous glucocorticoid excess. Lancet Diabetes Endocrinol..

[231] Pijls B.G., Jolani S., Atherley A., Derckx R.T., Dijkstra J.I.R., Franssen G.H.L., Hendriks S., Richters A., Venemans-Jellema A., Zalpuri S. (2021). Demographic risk factors for COVID-19 infection, severity, ICU admission and death: A meta-analysis of 59 studies. BMJ Open.

[232] Xu J., He L., Zhang Y., Hu Z., Su Y., Fang Y., Peng M., Fan Z., Liu C., Zhao K. (2021). Severe Acute Respiratory Syndrome Coronavirus 2 and Male Reproduction: Relationship, Explanations, and Clinical Remedies. Front. Physiol..

[233] Lisco G., Giagulli V.A., De Pergola G., De Tullio A., Guastamacchia E., Triggiani V. (2021). Covid-19 In Man: A Very Dangerous Affair. Endocr. Metab. Immune Disord. Drug Targets.

[234] Li D., Jin M., Bao P., Zhao W., Zhang S. (2020). Clinical Characteristics and Results of Semen Tests Among Men With Coronavirus Disease 2019. JAMA Netw. Open.

[235] Pan F., Xiao X., Guo J., Song Y., Li H., Patel D.P., Spivak A.M., Alukal J.P., Zhang X., Xiong C. (2020). No evidence of severe acute respiratory syndrome–coronavirus 2 in semen of males recovering from coronavirus disease 2019. Fertil. Steril..

[236] Gacci M., Coppi M., Baldi E., Sebastianelli A., Zaccaro C., Morselli S., Pecoraro A., Manera A., Nicoletti R., Liaci A. (2021). Semen impairment and occurrence of SARS-CoV-2 virus in semen after recovery from COVID-19. Hum. Reprod..

[237] Ruan Y., Hu B., Liu Z., Liu K., Jiang H., Li H., Li R., Luan Y., Liu X., Yu G. (2021). No detection of SARS-CoV-2 from urine, expressed prostatic secretions, and semen in 74 recovered COVID-19 male patients: A perspective and urogenital evaluation. Andrology.

[238] Best J.C., Kuchakulla M., Khodamoradi K., Lima T.F.N., Frech F.S., Achua J., Rosete O., Mora B., Arora H., Ibrahim E. (2021). Evaluation of SARS-CoV-2 in human semen and effect on total sperm number: A prospective observational study. World J. Mens. Health.

[239] Ma L., Xie W., Li D., Shi L., Ye G., Mao Y., Xiong Y., Sun H., Zheng F., Chen Z. (2021). Evaluation of sex-related hormones and semen characteristics in reproductive-aged male COVID-19 patients. J. Med. Virol..

[240] Song C., Wang Y., Li W., Hu B., Chen G., Xia P., Wang W., Li C., Diao F., Hu Z. (2020). Absence of 2019 novel coronavirus in semen and testes of COVID-19 patients. Biol. Reprod..

[241] Paoli D., Pallotti F., Colangelo S., Basilico F., Mazzuti L., Turriziani O., Antonelli G., Lenzi A., Lombardo F. (2020). Study of SARS-CoV-2 in semen and urine samples of a volunteer with positive naso-pharyngeal swab. J. Endocrinol. Investig..

[242] Kayaaslan B., Korukluoglu G., Hasanoglu I., Kalem A.K., Eser F., Akinci E., Guner R. (2020). Investigation of SARS-CoV-2 in Semen of Patients in the Acute Stage of COVID-19 Infection. Urol. Int..

[243] Holtmann N., Edimiris P., Andree M., Doehmen C., Baston-Buest D., Adams O., Kruessel J.S., Bielfeld A.P. (2020). Assessment of SARS-CoV-2 in human semen—A cohort study. Fertil. Steril..

[244] Gonzalez D.C., Khodamoradi K., Pai R., Guarch K., Connelly Z.M., Ibrahim E., Arora H., Ramasamy R. (2020). A systematic review on the investigation of sars-cov-2 in semen. Res. Rep. Urol..

[245] Bendayan M., Boitrelle F. (2021). COVID-19: Semen impairment may not be related to the virus. Hum. Reprod..

[246] Pozzilli P., Lenzi A. (2020). Testosterone, a key hormone in the context of COVID-19 pandemic. Metabolism.

[247] Mohamed M.S., Moulin T.C., Schiöth H.B. (2021). Sex differences in COVID-19: The role of androgens in disease severity and progression. Endocrine.

[248] Çayan S., Uğuz M., Saylam B., Akbay E. (2021). Effect of serum total testosterone and its relationship with other laboratory parameters on the prognosis of coronavirus disease 2019 (COVID-19) in SARS-CoV-2 infected male patients: A cohort study. Aging Male.

[249] Rastrelli G., Di Stasi V., Inglese F., Beccaria M., Garuti M., Di Costanzo D., Spreafico F., Greco G.F., Cervi G., Pecoriello A. (2021). Low testosterone levels predict clinical adverse outcomes in SARS-CoV-2 pneumonia patients. Andrology.

[250] Dhindsa S., Zhang N., McPhaul M.J., Wu Z., Ghoshal A.K., Erlich E.C., Mani K., Randolph G.J., Edwards J.R., Mudd P.A. (2021). Association of Circulating Sex Hormones With Inflammation and Disease Severity in Patients With COVID-19. JAMA Netw. Open.

[251] Bhasin S., Brito J.P., Cunningham G.R., Hayes F.J., Hodis H.N., Matsumoto A.M., Snyder P.J., Swerdloff R.S., Wu F.C., Yialamas M.A. (2018). Testosterone Therapy in Men with Hypogonadism: An Endocrine Society. J. Clin. Endocrinol. Metab..

[252] Scully E.P., Haverfield J., Ursin R.L., Tannenbaum C., Klein S.L. (2020). Considering how biological sex impacts immune responses and COVID-19 outcomes. Nat. Rev. Immunol..

[253] Marina S., Piemonti L. (2020). Gender and Age Effects on the Rates of Infection and Deaths in Individuals with Confirmed SARS-CoV-2 Infection in Six European Countries. SSRN Electron. J..

[254] Chen G., Wu D., Guo W., Cao Y., Huang D., Wang H., Wang T., Zhang X., Chen H., Yu H. (2020). Clinical and immunological features of severe and moderate coronavirus disease 2019. J. Clin. Investig..

[255] Mehta P., McAuley D.F., Brown M., Sanchez E., Tattersall R.S., Manson J.J. (2020). COVID-19: Consider cytokine storm syndromes and immunosuppression. Lancet.

[256] Fish E.N. (2008). The X-files in immunity: Sex-based differences predispose immune responses. Nat. Rev. Immunol..

[257] Klein S.L., Flanagan K.L. (2016). Sex differences in immune responses. Nat. Rev. Immunol..

[258] Roved J., Westerdahl H., Hasselquist D. (2017). Sex differences in immune responses: Hormonal effects, antagonistic selection, and evolutionary consequences. Horm. Behav..

[259] Al-Lami R.A., Urban R.J., Volpi E., Algburi A.M.A., Baillargeon J. (2020). Sex Hormones and Novel Corona Virus Infectious Disease (COVID-19). Mayo Clin. Proc..

[260] Phiel K.L., Henderson R.A., Adelman S.J., Elloso M.M. (2005). Differential estrogen receptor gene expression in human peripheral blood mononuclear cell populations. Immunol. Lett..

[261] Robinson D.P., Klein S.L. (2012). Pregnancy and pregnancy-associated hormones alter immune responses and disease pathogenesis. Horm. Behav..

[262] Doria A., Iaccarino L., Arienti S., Ghirardello A., Zampieri S., Rampudda M.E., Cutolo M., Tincani A., Todesco S. (2006). Th2 immune deviation induced by pregnancy: The two faces of autoimmune rheumatic diseases. Reprod. Toxicol..

[263] Di Toro F., Gjoka M., Di Lorenzo G., De Santo D., De Seta F., Maso G., Risso F.M., Romano F., Wiesenfeld U., Levi-D’Ancona R. (2021). Impact of COVID-19 on maternal and neonatal outcomes: A systematic review and meta-analysis. Clin. Microbiol. Infect..

[264] Chen L., Li Q., Zheng D., Jiang H., Wei Y., Zou L., Feng L., Xiong G., Sun G., Wang H. (2020). Clinical Characteristics of Pregnant Women with Covid-19 in Wuhan, China. N. Engl. J. Med..

[265] Allotey J., Stallings E., Bonet M., Yap M., Chatterjee S., Kew T., Debenham L., Llavall A.C., Dixit A., Zhou D. (2020). Clinical manifestations, risk factors, and maternal and perinatal outcomes of coronavirus disease 2019 in pregnancy: Living systematic review and meta-analysis. BMJ.

[266] Bwire G.M., Njiro B.J., Mwakawanga D.L., Sabas D., Sunguya B.F. (2021). Possible vertical transmission and antibodies against SARS-CoV-2 among infants born to mothers with COVID-19: A living systematic review. J. Med. Virol..

[267] Li F., Lu H., Zhang Q., Li X., Wang T., Liu Q., Yang Q., Qiang L. (2021). Impact of COVID-19 on female fertility: A systematic review and meta-Analysis protocol. BMJ Open.

[268] Bestle D., Heindl M.R., Limburg H., van Lam van T., Pilgram O., Moulton H., Stein D.A., Hardes K., Eickmann M., Dolnik O. (2020). TMPRSS2 and furin are both essential for proteolytic activation of SARS-CoV-2 in human airway cells. Life Sci. Alliance.

[269] Collins P., Webb C.M., de Villiers T.J., Stevenson J.C., Panay N., Baber R.J. (2016). Cardiovascular risk assessment in women—An update. Climacteric.

[270] Pirhadi R., Sinai Talaulikar V., Onwude J., Manyonda I. (2020). Could Estrogen Protect Women From COVID-19?. J. Clin. Med. Res..

[271] Trenti A., Tedesco S., Boscaro C., Trevisi L., Bolego C., Cignarella A. (2018). Estrogen, angiogenesis, immunity and cell metabolism: Solving the puzzle. Int. J. Mol. Sci..

[272] Suba Z. (2020). Prevention and therapy of covid-19 via exogenous estrogen treatment for both male and female patients; An opinion paper. J. Pharm. Pharm. Sci..

[273] Dyall J., Coleman C.M., Hart B.J., Venkataraman T., Holbrook M.R., Kindrachuk J., Johnson R.F., Olinger G.G., Jahrling P.B., Laidlaw M. (2014). Repurposing of clinically developed drugs for treatment of Middle East respiratory syndrome coronavirus infection. Antimicrob. Agents Chemother..

[274] Vermillion M.S., Ursin R.L., Attreed S.E., Klein S.L. (2018). Estriol reduces pulmonary immune cell recruitment and inflammation to protect female mice from severe influenza. Endocrinology.

[275] Channappanavar R., Fett C., Mack M., Ten Eyck P.P., Meyerholz D.K., Perlman S. (2017). Sex-Based Differences in Susceptibility to Severe Acute Respiratory Syndrome Coronavirus Infection. J. Immunol..

[276] Seeland U., Coluzzi F., Simmaco M., Mura C., Bourne P.E., Heiland M., Preissner R., Preissner S. (2020). Evidence for treatment with estradiol for women with SARS-CoV-2 infection. BMC Med..

[277] Kyrou I., Karteris E., Robbins T., Chatha K., Drenos F., Randeva H.S. (2020). Polycystic ovary syndrome (PCOS) and COVID-19: An overlooked female patient population at potentially higher risk during the COVID-19 pandemic. BMC Med..

[278] Moradi F., Enjezab B., Ghadiri-Anari A. (2020). The role of androgens in COVID-19. Diabetes Metab. Syndr. Clin. Res. Rev..

[279] Subramanian A., Anand A., Adderley N.J., Okoth K., Toulis K.A., Gokhale K., Sainsbury C., O’Reilly M.W., Arlt W., Nirantharakumar K. (2021). Increased COVID-19 infections in women with polycystic ovary syndrome: A population-based study. Eur. J. Endocrinol..

[280] Torjesen I. (2021). Covid-19 will become endemic but with decreased potency over time, scientists believe. BMJ.

[281] Creech C.B., Walker S.C., Samuels R.J. (2021). SARS-CoV-2 Vaccines. JAMA.

[282] Abdool Karim S.S., de Oliveira T. (2021). New SARS-CoV-2 Variants—Clinical, Public Health, and Vaccine Implications. N. Engl. J. Med..

[283] COVID-19 Vaccines|European Medicines Agency. https://www.ema.europa.eu/en/human-regulatory/overview/public-health-threats/coronavirus-disease-covid-19/treatments-vaccines/covid-19-vaccines.

[284] Polack F.P., Thomas S.J., Kitchin N., Absalon J., Gurtman A., Lockhart S., Perez J.L., Pérez Marc G., Moreira E.D., Zerbini C. (2020). Safety and Efficacy of the BNT162b2 mRNA Covid-19 Vaccine. N. Engl. J. Med..

[285] Baden L.R., El Sahly H.M., Essink B., Kotloff K., Frey S., Novak R., Diemert D., Spector S.A., Rouphael N., Creech C.B. (2021). Efficacy and Safety of the mRNA-1273 SARS-CoV-2 Vaccine. N. Engl. J. Med..

[286] Voysey M., Clemens S.A.C., Madhi S.A., Weckx L.Y., Folegatti P.M., Aley P.K., Angus B., Baillie V.L., Barnabas S.L., Bhorat Q.E. (2021). Safety and efficacy of the ChAdOx1 nCoV-19 vaccine (AZD1222) against SARS-CoV-2: An interim analysis of four randomised controlled trials in Brazil, South Africa, and the UK. Lancet.

[287] European Society of Endocrinology (ESE)’s Statement Concerning COVID-19 Vaccination: ‘Follow the Same Recommendations for Patients with Stable Endocrine Disorders as for the General Population’|ESE. https://www.ese-hormones.org/news/ese-news/european-society-of-endocrinology-ese-s-statement-concerning-covid-19-vaccination-follow-the-same-recommendations-for-patients-with-stable-endocrine-disorders-as-for-the-general-population/.

[288] Katznelson L., Gadelha M. (2021). Glucocorticoid use in patients with adrenal insufficiency following administration of the COVID-19 vaccine: A pituitary society statement. Pituitary.

