# Supplementary material for: COVID-19 and the Endocrine System: A Comprehensive Review on the Theme
Source: J Clin Med. 2021 Jun 29;10(13):2920. doi: 10.3390/jcm10132920 (PMC8269331; doi:10.3390/jcm10132920)
Supplement: Supplementary file 1 [file jcm-10-02920-s001.zip › jcm-1198954-supplementary.pdf]

**Supplementary Table S1.** Summary of the main descriptive findings related to the three specific questions.

| | SARS-CoV-2 Mediated injury | Hypothesized Mechanisms of Worse Progression of COVID-19 (If Baseline Condition) | Management |
|---|---|---|---|
| Cushing's disease | | Hyperglycemia<br>Increase in arterial blood pressure<br>Thrombotic diathesis<br>High cardiovascular risk [61,62]<br>Pharmacological interferences [66–68] (hypoglycemia; QTc prolongation; hypokalemia)<br>- Dexamethasone and mifepristone<br>- Tocilizumab and ketoconazole and mifepristone<br>- Mitotane and Remdesivir<br>- Somatostatin analogues and hydroxychloroquine | Tight control of risk factors if sick<br>Avoid unnecessary in-person follow-up visits until fully vaccinated<br>Plan alternative strategy of follow-up visits (i.e., telemedicine)<br>Prioritize access to diagnosis and cure of CD cases in case of high suspicion only<br>Prioritize vaccine administration can be useful |
| Adrenal insufficiency | Hypophysitis due to cross-reacting antibodies against endogenous ACTH [34,37]<br>Acute bilateral adrenal thrombosis or hemorrhages (i.e., sepsis) [210,213] | Adrenal crisis may increase mortality in case of infectious disease, including COVID-19 [90]<br>Immune system imbalance (i.e., NK cells cytotoxicity and mononuclear cells impairment [206,207])<br>Hypotension<br>Arrhythmias (hypokalemia) | Supplement hydrocortisone replacement per os in case of signs and symptoms of systemic inflammation regardless of COVID-19 etiology (sick day rule) [78]<br>Consider hydrocortisone supplementation even in case of vaccine-related signs and symptoms (i.e., fever)<br>Emergency call or urgent access to the emergency department when an adrenal crisis is highly suspected (hypotension, vomiting, fatigue, abdominal pain)<br>Adequate steroidal tapering before discharge in case of severe COVID-19 cases treated with dexamethasone<br>Prioritize vaccine administration can be useful |
| Diabetes insipidus | Direct or immune-mediated (neuro)hypophysitis [51]<br>Hypoxic encephalopathy consequent to severe respiratory insufficiency [52]<br>Sevoflurane for endotracheal intubation (nephrogenic) [53] | Thrombotic diathesis<br>(severe hypernatremia)[56] | Adequate monitoring of diuresis, fluid intake, serum electrolytes, plasmatic and urinary osmolarity, arterial pressure<br>Supplementation of hypotonic fluids and desmopressin (orally, intranasal, or parenterally based upon clinical background conditions) [54]<br>Periodic and intermittent desmopressin withdrawal (i.e., a day per week for aquaresis) if access to laboratory examinations is difficult |
| Acromegaly | | High cardiovascular risk [70,71]<br>Background pulmonary complaints [70,71]<br>Hyperglycemia [70,71]<br>Increase in arterial blood pressure [70,71] | Achieve normal or near-normal IGF-1 levels<br>Consider additional treatments for controlling comorbidities (hyperglycemia or arterial hypertension), such as GH receptor antagonists<br>Pituitary surgery should be selected based upon clinical conditions and epidemiological pressure<br>Prioritize vaccine administration can be useful |
| GH Deficiency | | No general complaints | Avoid discontinuation of GH replacement |
| Hypopituitarism | Possible onset of hypophysitis and hypopituitarism [36] | Possible acute risks in case of adrenal insufficiency | Precocious recognition of risk factors suggestive for new-onset hormonal deficit (mostly hypoadrenalism and hypothyroidism) requiring urgent replacement treatment<br>Prioritize vaccine administration can be useful |
| Hypothyroidism | Subacute thyroiditis [82–89]<br>Atypical thyroiditis [82–89] | No general complaints<br>Cardiovascular events possibly due to severe and | Maintain adequate management of patients also at-distance |

| | | | |
|---|---|---|---|
| | Possible trigger of thyroid autoimmunity (long-term) | uncontrolled hypothyroidism [121,122] | Evaluate those patients with a medical history of SARS-CoV-2 infection complicated to thyroid dysfunction<br>Evaluate possible long-term thyroid complaints in those who recovered from COVID-19 |
| Hyperthyroidism | Possible trigger of thyroid autoimmunity (long-term)<br>Relapse or recurrence of previously controlled hyperthyroidisms | No general complaints<br>Cardiovascular events possibly due to severe and uncontrolled hyperthyroidism [121,122] | Guarantee adequate follow-up also at-distance<br>New-onset cases should be recognized and treated promptly<br>A block and replace regimen may reduce the need for repeated TSH measurement to titrate antithyroid medications<br>In case of occurrence of autoimmune hyperthyroidism in patients who recovered from COVID-19, long-term follow up could help acquire epidemiological information |
| Non-thyroidal illness syndrome | Related to a high-grade systemic inflammation [103,104]<br>Usually observed in seriously ill patients | It is unclear if it should be always considered as an adaptive mechanism rather than may have a role in worsening the prognosis of severe COVID-19 cases [111] | Further research will indicate if NTIS should be treated and which patients will obtain clinical benefits [116] |
| Thyroid nodule | - | No reasonable risks | Assess nodule risk stratification<br>Prioritize fine needle aspiration and cytology for at-risk population only [129,130] |
| Thyroid carcinoma | - | No reasonable risks | Ensure adequate follow-up also at-distance<br>Prioritize surgery based upon clinical staging and airways complaints[131,132]<br>Access to radioactive iodine therapy based upon initial risk stratification |
| Hypercalcemia | | No reasonable risks | Prioritize patients requiring parathyroidectomy<br>Medical management should be started or continued in mild forms or in those case in which surgery is declined or contraindicated |
| Hypocalcemia | Related to a high-grade systemic inflammation | Possible role of hypocalcemia in worsen the prognosis, but it could be necessary to avoid confounders (e.g., renal insufficiency, hypoalbuminemia) [165–167] | Calcium, Vitamin D, and where necessary, Magnesium supplementations are necessary to maintain adequate calcium homeostasis<br>Follow-up should also be guaranteed at-distance by telemedicine<br>Reinforce education to recognize and manage symptoms of latent hypocalcemia |
| Primary Osteoporosis | Systemic inflammation [199]<br>Prolonged use of systemic glucocorticoids [199]<br>Prolonged bed immobilization of severe cases [199]<br>Prolonged home self-confinement [199] | No reasonable risks | Prevent with vitamin D and calcium supplementation in high-risk patients<br>Discontinuation of antiresorptive medications (i.e., denosumab) is not recommended as it may increase the short-term risk of fracture considerably [184]<br>Prioritize vaccine administration can be useful |
| Cushing syndrome | | Cardiovascular risk [221,222]<br>Thrombotic diathesis [223]<br>Immunosuppression, and background systemic inflammation [224]<br>Increased risk of opportunistic infections | Control risk factors through specific therapies<br>Provide antibiotics/antimycotic therapy to prevent respiratory superinfections [231]<br>Prioritize vaccine administration can be useful |
| Male hypogonadism | Direct or immune-coagulative mediated testicular damage [233,234]<br>Sperm quantitative and qualita- | Testosterone may foster SARS-CoV-2 internalization into host cells [180]<br>Testosterone deficiency increases the risk of cardiovascular events; may precipitate thrombotic events | Testosterone replacement therapy should be continued in case of SARS-CoV-2 infection [252]<br>Long-term follow up could provide help- |

| | | | |
|---|---|---|---|
| | tive changes (due to SARS-CoV-2 or instead to fever, concomitant use of antibiotics/systemic steroids) | and exacerbates immune system dysfunction; may worsen glucose control and foster weight gain and visceral adipose tissue accumulation [180,248]<br>Patients displaying low levels of Testosterone, despite confounding factors, exhibit poor prognosis due to COVID-19 | ful information about testicular heath in patients who had recovered from COVID-19<br>Testosterone assessment in hospitalized COVID-19 patients may have a prognostic role<br>Prioritize vaccine administration can be useful, especially when T deficiency in elderly and comorbid patients |
| Polycystic ovary syndrome | Despite ovarian tissue express ACE2, it has been excluded a possible SARS-CoV-2 related ovarian damage | Increased risk of contracting SARS-CoV-2 [280]<br>Androgens may predispose to a greater cardiovascular risk than the general population | Search for reasons explaining a possible significant risk of contracting SARS-CoV-2 and reinforce hygienic tips to decline this burden<br>Prioritize vaccine administration can be useful |
